# Telemetry and genetics reveal asymmetric dispersal of a lake‐feeding salmonid between inflow and outflow spawning streams at a microgeographic scale

**DOI:** 10.1002/ece3.5937

**Published:** 2020-02-07

**Authors:** Ross Finlay, Russell Poole, Jamie Coughlan, Karl P. Phillips, Paulo Prodöhl, Deirdre Cotter, Philip McGinnity, Thomas E. Reed

**Affiliations:** ^1^ School of Biological, Earth and Environmental Sciences University College Cork Cork Ireland; ^2^ Marine Institute Furnace Newport Ireland; ^3^ Institute for Global Food Security School of Biological Sciences Queen's University Belfast Belfast Ireland

**Keywords:** dispersal, lake, local adaptation, philopatry, sympatric populations, telemetry

## Abstract

The degree of natal philopatry relative to natal dispersal in animal populations has important demographic and genetic consequences and often varies substantially within species. In salmonid fishes, lakes have been shown to have a strong influence on dispersal and gene flow within catchments; for example, populations spawning in inflow streams are often reproductively isolated and genetically distinct from those spawning in relatively distant outflow streams. Less is known, however, regarding the level of philopatry and genetic differentiation occurring at microgeographic scales, for example, where inflow and outflow streams are separated by very small expanses of lake habitat. Here, we investigated the interplay between genetic differentiation and fine‐scale spawning movements of brown trout between their lake‐feeding habitat and two spawning streams (one inflow, one outflow, separated by <100 m of lake habitat). Most (69.2%) of the lake‐tagged trout subsequently detected during the spawning period were recorded in just one of the two streams, consistent with natal fidelity, while the remainder were detected in both streams, creating an opportunity for these individuals to spawn in both natal and non‐natal streams. The latter behavior was supported by genetic sibship analysis, which revealed several half‐sibling dyads containing one individual that was sampled as a fry in the outflow and another that was sampled as fry in the inflow. Genetic clustering analyses in conjunction with telemetry data suggested that asymmetrical dispersal patterns were occurring, with natal fidelity being more common among individuals originating from the outflow than the inflow stream. This was corroborated by Bayesian analysis of gene flow, which indicated significantly higher rates of gene flow from the inflow into the outflow than vice versa. Collectively, these results reveal how a combination of telemetry and genetics can identify distinct reproductive behaviors and associated asymmetries in natal dispersal that produce subtle, but nonetheless biologically relevant, population structuring at microgeographic scales.

## Graphical abstract

We used a combination of PIT telemetry and genetic analyses to investigate the interplay between fine‐scale spawning movements and population structure of brown trout within a complex river system. Our research revealed evidence of distinctly asymmetrical, downstream‐biased dispersal patterns between inflow and outflow streams separated by less than 100 m of lake habitat. This asymmetry appears to facilitate the occurrence and maintenance of genetic structure at a microgeographic scale.

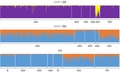

## 1 INTRODUCTION

Natal philopatry, whereby individuals return to their birth place for reproduction, limits gene flow between geographic areas and thereby increases neutral genotypic differentiation among populations via genetic drift. When ecological conditions vary across space, natal philopatry can also facilitate the evolution of local adaptation (Kawecki & Ebert, 2004), which in turn influences the resilience of metapopulations and species in the face of environmental change (Hilborn, Quinn, Schindler, & Rogers, 2003; Schindler, Armstrong, & Reed, 2015). However, the geographic scales over which local adaptation operates within salmonid species remain poorly characterized (Adkison, 1995; Fraser, Weir, Bernatchez, Hansen, & Taylor, 2011). In contrast to philopatry, natal dispersal promotes gene flow, increasing genetic diversity and thus reducing the likelihood of inbreeding within populations and homogenizing genetic structure among populations, sometimes at the expense of local adaptation (Garant, Forde, & Hendry, 2007). Rates of philopatry versus dispersal can vary within a single species with respect to sex, age, life history, or environmental factors (De Fraipont, Clobert, John, Alder, & Meylan, 2000; Förschler, Val, & Bairlein, 2010; Lesage, Crête, Huot, Dumont, & Ouellet, 2000; Purdue, Smith, & Patton, 2000; Winkler et al., 2006). At the individual level, these behaviors are associated with a range of context‐dependent fitness consequences, with many theories having been proposed for when selection should favor philopatry over dispersal, or vice versa (see review by Hendry, Castric, Kinnison, & Quinn, 2004).

Dispersal is distinct from migration, in a behavioral sense, with the latter corresponding to spatially and temporally predictable movement of individuals among breeding and foraging or refuge habitats (Dingle & Drake, 2007). However, dispersal and migration can be related. For example, resident passerine birds exhibit higher rates of natal philopatry (i.e., reduced natal dispersal) relative to migratory passerine birds (Weatherhead & Forbes, 1994). Similarly, genetic differentiation appears to be greater among lake‐ and stream‐resident populations of brown trout (*Salmo trutta*) compared with anadromous (sea‐migrating) populations (Östergren & Nilsson, 2012), implying that “straying” (i.e., natal dispersal) rates may be higher in the latter, perhaps due to constraints on homing abilities.

Brown trout in general exhibit a broad range of migratory strategies and distances (Ferguson, Reed, Cross, Mcginnity, & Prodöhl, 2019; Nevoux et al., 2019), making them a particularly interesting species for studying links between movement behavior, dispersal versus philopatry, and the extent of demographic and genetic connectedness of populations.

Natal philopatry in salmonids involves a complex interaction between evolved genetic mechanisms and proximal responses to environmental and social cues (Dittman & Quinn, 1996). Together these mechanisms allow salmonids to “home” back to their natal river and even the natal sites from which they originated, despite intervening movements or feeding migrations that can range in extent from tens of meters to thousands of kilometers (Neville, Isaak, Dunham, Thurow, & Rieman, 2006; Quinn, Stewart, & Boatright, 2006; Stewart, Quinn, & Bentzen, 2003). The geographic scale and consistency at which such homing behavior operates remain uncertain, although individual Atlantic salmon have been recorded breeding in multiple redds separated by distances ranging from less than five meters up to more than five kilometers (Taggart, McLaren, Hay, Webb, & Youngson, 2001). Juvenile salmonids imprint on (i.e., learn) the odors of their natal stream prior to, or during, out‐migration from it (Dittman & Quinn, 1996; Keefer & Caudill, 2014). However, interrupted or imperfect imprinting during rearing or juvenile migration can increase straying rates (Keefer & Caudill, 2014). There also appear to be genetically based differences across populations in straying rates and distances (Jonsson & Jonsson, 2014, 2017; Keefer & Caudill, 2014; King, Hillman, Elsmere, Stockley, & Stevens, 2016), with selection thought to favor higher straying when habitat quality or quantity fluctuates unpredictably through time (Hendry et al., 2004; Quinn & Tallman, 1987).

In addition to behavioral and life‐history characteristics, landscape or seascape features also play a strong role in promoting or limiting dispersal and thus shaping patterns of intraspecific genetic diversity across space. For example, population structure in freshwater fishes tends to greatly exceed that found in marine fishes, perhaps due to the presence of more physical barriers to dispersal within and among freshwater systems (Tonteri, Veselov, Titov, Lumme, & Primmer, 2007; Ward, Woodwark, & Skibinski, 1994). Waterfalls, culverts, dams, and other landscape features can obstruct movement within river systems in both directions or in just one, with the latter situation providing a mechanism for asymmetric dispersal and gene flow (Prodőhl et al., 2019; Torterotot, Perrier, Bergeron, & Bernatchez, 2014). Asymmetric dispersal, which also occurs in terrestrial habitats where it is often wind‐driven (Cook & Crisp, 2005; Sanmartı, Wanntorp, & Winkworth, 2007), and in marine environments where it is often driven by ocean currents (Pringle, Blakeslee, Byers, & Roman, 2011; Storch & Pringle, 2018), can effectively generate a source‐pseudosink population structure (sensu Watkinson & Sutherland, 1995) in which natural selection should be biased in favor of the source (“upstream”) habitat (Kawecki & Holt, 2002). Additionally, river characteristics can interact with homing and life‐history differences to influence the genetic diversity and structure of salmonid populations (Bradbury et al., 2013; Gomez‐Uchida, Knight, & Ruzzante, 2009; Mcphee, Whited, Kuzishchin, & Stanford, 2014; Ozerov, Veselov, Lumme, & Primmer, 2012; Vähä, Erkinaro, Niemelä, & Primmer, 2007), as may the presence of lakes within watersheds (Dillane et al., 2008; Jacobs, Hughes, Robinson, Adams, & Elmer, 2018; Massa‐Gallucci, Coscia, O'Grady, Kelly‐Quinn, & Mariani, 2010; McKeown, Hynes, Duguid, Ferguson, & Prodöhl, 2010; Palmé, Laikre, & Ryman, 2013). In addition to isolation‐by‐dispersal limitation, so‐called “isolation‐by‐adaptation” processes may serve to increase genome‐wide differentiation among populations, where natural selection plays an indirect role by reducing gene flow among ecologically divergent habitats, owing to reduced fitness of immigrants (Nosil, Egan, & Funk, 2008; Orsini, Vanoverbeke, Swillen, Mergeay, & Meester, 2013). With reduced gene flow, populations are “free” to diverge under the influence of random genetic drift. Isolation by adaptation has been invoked to explain spatial patterns of genetic diversity in salmonids linked with, for example, climate (Dionne, Caron, Dodson, & Bernatchez, 2008; Hand et al., 2016; Olsen et al., 2011), geological substrate (Perrier, Guyomard, Bagliniere, & Evanno, 2011), pathogens (de Eyto et al., 2011), and metal contamination (Paris, King, & Stevens, 2015).

In this study, we use a combination of telemetry and genetics to investigate the interplay between putative homing/straying behaviors and genetic differentiation among spawning streams at a microgeographic scale (sensu Richardson, Urban, Bolnick, & Skelly, 2014) in nonanadromous brown trout. The species typically exhibits hierarchical population genetic structure across a range of spatial scales (Lobón‐Cerviá & Sanz, 2017), sometimes down to scales of <1 km (Carlsson, Olsén, Nilsson, Øverli, & Stabell, 1999), implying either low straying rates at these microgeographic scales or fine‐scale local adaptation that constrains gene flow if straying does occur. Brown trout often exploit lakes for growing and rearing, which can involve short‐ or long‐distance migrations between natal streams and lacustrine habitat. A particularly interesting scenario arises where brown trout spawn in both inflowing and outflowing streams, but co‐occur in a more productive lake habitat for much of their lives. Juveniles spawned in lake‐outflows must conduct upstream feeding migrations to reach lake habitat, actively swimming against the flow of the river. Such upstream feeding migrations would presumably be maladaptive in lake‐inflow streams, as this behavior would move juveniles away from, rather than toward, the lake, where growth opportunities are higher. Thus, inflowing and outflowing streams may exert differing selective pressures by virtue of their flow direction, which in turn could promote genome‐wide genetic divergence via the above mechanisms. Indeed, Jonsson, Jonsson, Skurdal, and Hansen (1994) demonstrated that the offspring of inflow and outflow spawning brown trout displayed different directional migratory responses to water current, a population‐specific juvenile rheotactic response pattern that has been identified in various salmonid species (Bowler, 1975; Brannon, 1972; Kaya, 1991; Kelso & Northcote, 1981; Raleigh, 1967, 1971; Raleigh & Chapman, 1971). Additionally, brown trout populations that utilize common lake‐feeding habitat but are genetically, behaviorally and morphologically distinct have been found to display reproductive isolation by homing back to separate inflow or outflow rivers for spawning (Ferguson & Mason, 1981; Ferguson & Taggart, 1991; Jacobs et al., 2018). Reproductive isolation appears to promote similar differentiation among sympatric lake‐dwelling populations of rainbow smelt (*Osmerus mordax*) (Taylors & Bentzent, 1993), Arctic charr (*Salvelinus alpinus* L.) (Jonsson & Jonsson, 2001), Dolly Varden (*Salvelinus malma*) (Markevich, Esin, & Anisimova, 2018), and sockeye salmon (*Oncorhynchus Nerka*) (Moreira & Taylor, 2015). It remains unknown, however, whether consistent, accurate homing behavior and associated genetic divergence occurs between lake‐inflow and lake‐outflow streams at microgeographic scales of less than 100 m.

Here, we investigated these issues in a small lake in the west of Ireland that is fed and drained respectively by a single inflowing and a single outflowing stream, separated by less than a hundred meters of lake habitat. We hypothesized that a combination of pre‐ and postzygotic isolating mechanisms should have produced weak to moderate neutral genetic differentiation between brown trout originating in the inflow and outflow streams, and that gene flow patterns between these groups may not be symmetrical. As our first aim, we used PIT‐tag telemetry to monitor lake‐to‐stream movements of spawning‐sized trout to determine whether some fish exhibited behavior consistent with philopatry (only detected in one of the streams) while the behavior of others was consistent with straying (detected in both streams). A fish detected during the spawning season in only one of the streams may, of course, have been born in the other and thus have been exhibiting straying behavior. In the absence of more direct methods for detecting homing versus straying, genetic techniques can be used to assign fish sampled as adults in the lake to population genetic clusters that may correspond to inflow versus outflow spawning streams if gene flow is restricted. Similarly, parental movements may be inferred indirectly using genetic sibship analysis: if fry sampled in both streams assign to the same half‐sib group, for example, this suggests that one of their parents spawned in both streams. Our second aim was therefore to use a range of genetic analyses, including clustering approaches, to identify any such half‐sibships, test for fine‐scale population structure, characterize patterns of gene flow between the streams (symmetric vs. asymmetric), and interpret these patterns in light of the behavioral data and vice versa.

## 2 MATERIALS AND METHODS

### 2.1 Study area

**Figure A1:**
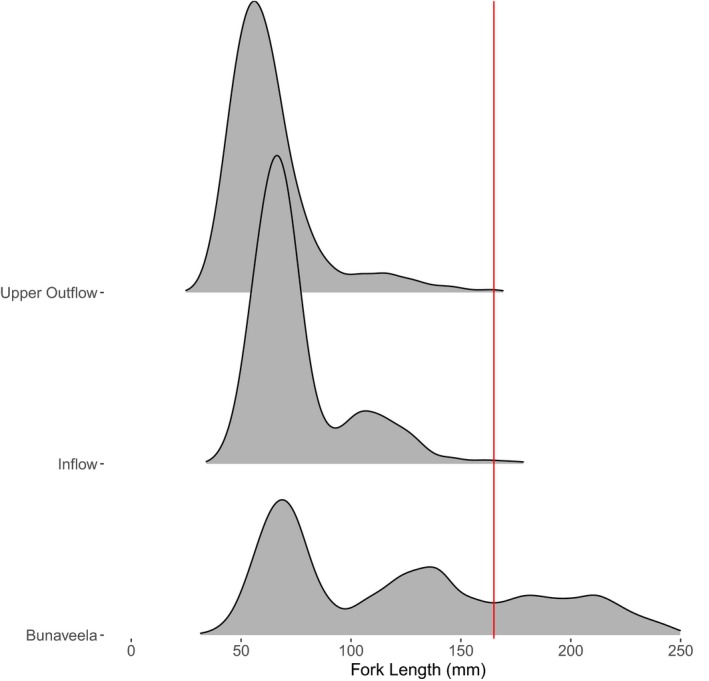
Size distribution (FL) of trout sampled in the inflow (*n* = 877), the upper outflow (*n* = 1,136), and Bunaveela Lough (*n* = 3,176). The red line at 165 mm represents the threshold length for maturity selected for this study (mean – 1*SD*). Sampling period: June–September 1991–2017

The Burrishoole catchment in the northwest of Ireland is a complex freshwater system comprised of three main lakes linked by a network of rivers and streams that drain an area of approximately 83km^2^. Bunaveela Lough (54º01′18″ N 9º32′43″ W), the most northerly and the most elevated of the three lakes, has a surface area of 46 ha, a maximum depth of 23 m, and supports populations of brown trout (*Salmo trutta* L.), Atlantic salmon (*Salmo salar* L.), Arctic char (*Salvelinus alpinus* L.), and European eel (*Anguilla anguilla* L.). Seine netting surveys indicate that trout are relatively abundant in the lake, outnumbering salmon and char within the littoral zone by more than five to one. The lake is fed by a single inflowing stream, the Fiddaunveela, and drained by a single outflowing stream, the Goulaun. The straight line distance between the point at which the Fiddaunveela flows into the lake and the point at which the Goulaun flows out of the lake is 98 m. The inflow is a shallow and flood‐prone stream of approximately 2,010 m in length, draining a steep valley to the southeast of the lake. The outflow flows southwest from Bunaveela Lough for 10,345 m before entering Lough Feeagh (410 ha), increasing significantly in size as it approaches Feeagh. Due to the regulating effect of Bunaveela, the upper stretches of the outflow are less prone to rapid fluctuations in flow rates than the inflow. Although pH during baseflow is circumneutral in the two streams, they are both small, poorly buffered and oligotrophic, and therefore offer limited feeding and growth opportunities to resident trout (hydrological conditions described in Appendix Table A1). In contrast, much of Bunaveela Lough is comparatively well buffered and productive due to limestone and sandstone deposits (Whelan, Poole, McGinnity, Rogan, & Cotter, 1998) and consequently the lake represents an alternative feeding habitat where growth may be less constrained (trout size distributions in streams and lake shown in Appendix Figure A1).

### 2.2 Sampling

**Figure 1:**
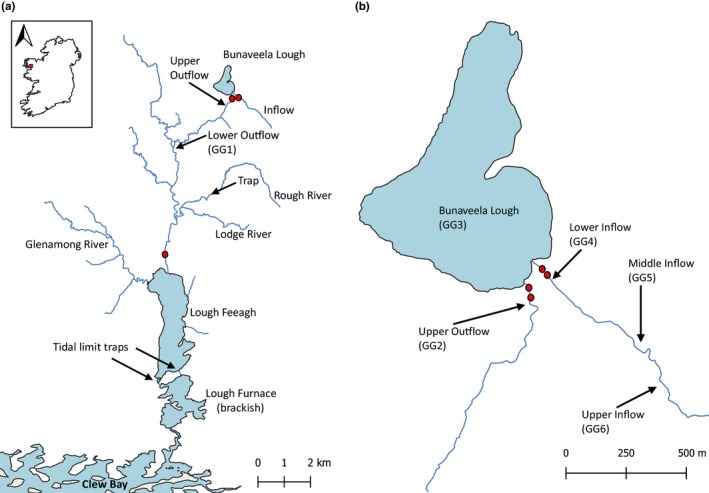
(a) Map of the Burrishoole catchment, Co. Mayo, Ireland. (b) Expanded view of Bunaveela Lough and the upper Burrishoole catchment. Red circles are locations of PIT antennae. GG refers to geographic groups, that is, sites where groups of fish were sampled

Trout fry and parr were captured during the summers of 2017 and 2018 by electrofishing (electrofisher model: Hans Grassl IG600) in the upper and lower sections of the outflow (*n* = 181) and the upper, middle, and lower sections of the inflow (*n* = 208) (Figure 1). A total of 500 additional trout (fry, parr, and adults) were captured from the southeastern shore of Bunaveela Lough (henceforth “Bunaveela”) by seine netting (9‐mm mesh) across six dates between October 2016 and October 2018 (details in Appendix Table A2). All trout were anaesthetized in pH‐buffered tricaine methane sulfonate (80 mg/L), measured (fork length, FL, mm), and weighed (to 0.1 g), and a small clip (*c*. 2 mm^2^) was taken from the caudal fin and stored in 95% ethanol for genetic analysis. Each trout of >70 mm (*n* = 605) was implanted with a uniquely coded 12 mm half‐duplex (HDX) passive integrated transponder (PIT) tag (Biomark 134.2 kHz ISO HDX). PIT tags were implanted into the peritoneal body cavity through a needle inserted just posterior to the tip of the pectoral fin and to one side of the mid‐ventral line at the tips of the pleural ribs. After sampling, anaesthetized fish were moved to a tank of aerated fresh river or lake water and monitored until their equilibrium was fully regained and active swimming recommenced. Once recovered, all fish were released back into the site from which they were originally captured. PIT tagging and fin clipping were carried out in accordance with S.I.No. 123/2014 Animal Health and Welfare (operations and procedures) Regulations 2014 and with approval of the MI animal welfare committee.

In order to characterize movement patterns at a broader catchment scale and thus contextualize any fine‐scale genetic structure observed during subsequent analysis, a combination of electrofishing and seine netting was used to capture trout of a range of sizes in Lough Feeagh and three of its tributaries (the Rough River, the Lodge River, and the Glenamong River) (Figure 1) over 32 dates between October 2016 and October 2018 (Appendix Table A2). Additionally, between December 2016 and March 2019 one fish trap was operated continuously in the Rough River (RR), a river that is utilized by lake‐feeding trout for spawning, and two other traps were operated at tidal limit of the river system. Trout that had been actively captured or trapped in either of the above cases were measured, visually assessed for maturity status, scanned for PIT tags, and all untagged individuals >70 mm (*n* = 3,617) were PIT tagged. A length distribution for trout confirmed as mature (i.e., displaying physical characteristics indicative of imminent or recent mating) was generated and used to designate a minimum threshold “mature” length (mean FL minus 1 *SD* of mature fish) for use in behavioral analyses. A conservative threshold of one standard deviation below the mean was used here in order to maximize the chances of only including true mature fish in the telemetry analyses (as we were interested in spawning movement behaviors specifically), that is, to exclude larger immature fish that may have been similar in size to relatively small mature fish.

Most of the trout that were PIT‐tagged in Bunaveela (87.6%) were only caught once (up to 811 days prior to the spawning period during which they were detected on an antenna), and therefore, their actual size at spawning time was unknown. To estimate this, and thereby exclude any individuals that were below our threshold mature length from subsequent analyses, we used FL data from 87 PIT‐tagged lake‐feeding trout that were recaptured during the study period (from 15 to 505 days after tagging) to calibrate a linear model to describe growth per degree day in the statistical program R v3.5.2 (R Core Team, 2018). This then allowed us to infer the likely growth (from tagging to spawning period) of fish that were only measured at tagging, giving an estimate of their FL during the spawning period. Previous studies have shown that individual growth rate within fish populations is largely a function of temperature and individual size (Boltaña et al., 2017; Handeland, Imsland, & Stefansson, 2008; Neuheimer & Taggart, 2007). Throughout the study period, high frequency lake surface temperature data were recorded in Lough Feeagh (~7 km SW of Bunaveela) (de Eyto et al., 2019) and, due to the physical similarities and geographical proximity of the lakes, these temperatures were used as a proxy for Bunaveela surface water temperatures. By including a base temperature (*T*_0_), calculations were limited to temperatures relevant to growth, or “growing degree days” (GDD) (Chezik, Lester, & Venturelli, 2014). FL at tagging date and GDD between tagging date and recapture date were treated as explanatory variables while growth over the same period was treated as the response variable in the model. The best value for *T*_0_, in statistical terms, was determined by maximizing model *R* ^2^ value from a range of base temperatures (0°C–12°C inclusive). The model containing the optimized *T*_0_ was then used to estimate individual growth since tagging (and thus final FL) as a function of initial FL and intervening GDD.

### 2.3 Monitoring behavior—PIT telemetry

In order to investigate the movement of lake‐feeding trout during the spawning season, as well as the degree of movement between the upper and lower Burrishoole catchment, a network of five swim‐through, cross‐channel HDX PIT antennae powered by Oregon RFID multiplexing readers was installed between August and September 2017 and maintained for the duration of the study period. HDX PIT antennae generate an electromagnetic field that wirelessly powers any nearby HDX PIT tag, causing the tag to transmit a unique 12‐digit identification number that is subsequently received and recorded by the antenna reader along with the date and time. The scan rates of all readers were set to ten transmit–receive cycles/s. Two antennae were installed in the lower inflow, 75 and 85 m upstream of Bunaveela, and two antennae were installed in the upper outflow, 40 and 60 m downstream of the lake (Figure 1). A single antenna was installed in the lower outflow, 9,540 m downstream from Bunaveela and 805m upstream from Lough Feeagh (Figure 1). Each antenna was designed to span the entire channel width and depth at its location during all but exceptionally high flow conditions. The performance of each antenna was checked with a test tag every 10–14 days and shortly after each flood event. When required, repairs were generally completed within 48 hr of antenna damage in order to ensure that antennae remained operational throughout the vast majority study period.

**Figure A2:**
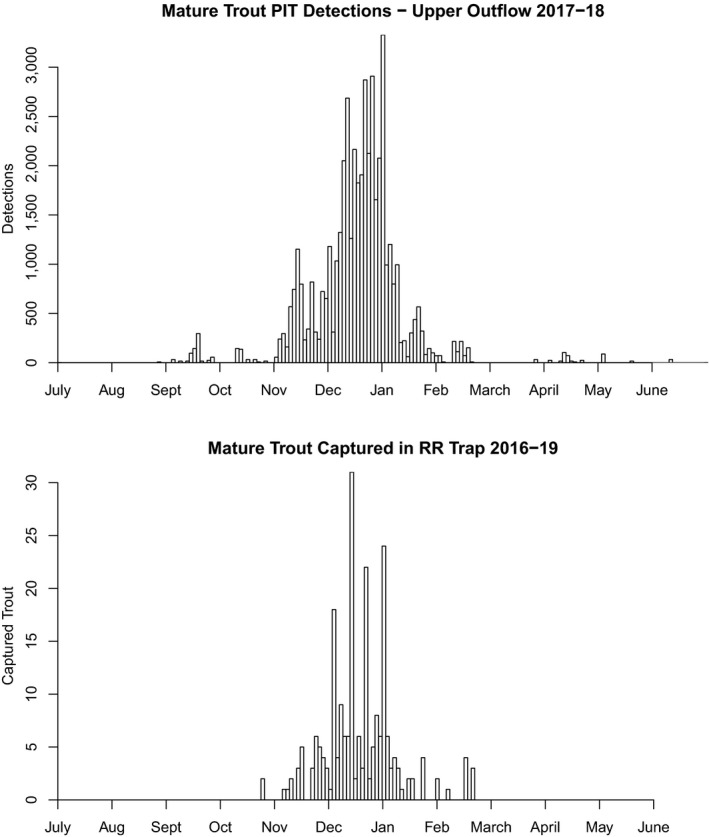
Temporal distribution of PIT detections from mature‐sized Bunaveela‐tagged trout moving past the upper outflow antennae (July 2017 to June 2018) and phenotypically mature trout (i.e., gravid or ripe) captured at the Rough River fish trap during the study period

A combination of daily data on the maturity status of trout moving upstream and downstream through the RR fish traps and PIT‐derived behavioral data for Bunaveela‐tagged trout deemed mature was used to designate a spawning migration period for Burrishoole trout, running from the 1st of November to the 28th of February. Annual movements of mature‐sized lake‐tagged trout past the fluvial antennae occurred almost exclusively during this period (see results and Appendix Figure A2), suggesting that movements recorded at this time of the year were primarily motivated by reproduction rather than exploratory foraging. For the purpose of characterizing discrete behavioral tactics, mature trout detected only on the inflow antennae were categorized simply as “inflow‐only” (IO) fish, and mature fish detected only on the upper outflow antennae as “outflow‐only” (OO) fish. Fish from each of these categories could be exhibiting homing behavior to their natal streams, or straying behavior if born in the other stream—the telemetry data alone cannot distinguish these, but genetic inference may facilitate such distinctions (see below). Mature trout detected on antennas in both streams during the spawning window were categorized simply as B fish (standing for “both”), which could be exhibiting either homing or straying, or both; clearly they moved from the lake to both streams, but they may not have spawned in them.

### 2.4 Genetics

#### 2.4.1 DNA extraction

Genomic DNA was extracted from caudal tissue from 853 samples using the Promega Wizard® SV 96 Genomic DNA Purification System. DNA quality and quantity were assessed on agarose gels by comparison with a Quick‐Load® Purple 100 bp DNA Ladder (New England Biolabs). Concentration of DNA for PCR was adjusted to 2–10 ng/µl.

#### 2.4.2 PCR

Multiplex PCR (two independent reactions) was used to amplify 18 microsatellite loci (1—Ssa197; 2—Ssa85; 3—SsaD71; 4—Ssa410UOS; 5—Ssa416; 6—CAO48828; 7—CAO53293; 8—CAO60177; 9—BG935488; 10—One102‐a; 11—One103; 12—One108; 13—ppStr3; 14–One102‐b; 15—Cocl‐lav‐4; 16—SasaTAP2A; 17—MHC‐I; and 18—One9Asc) in addition to one sex marker, which was developed from the SalmoYF sequence available in GenBank (P. Prodöhl, unpublished). These microsatellites, selected from a panel comprising 38 markers characterized and optimized by Keenan, Bradley, et al. (2013) for *Salmo trutta* genetic research, had been found to be very reliable, consistent, and informative for population genetic structuring (Prodöhl et al., 2017). All PCRs were performed in 3.5 µl volume, including ~2–10 ng of genomic DNA and 1.75 µl Plain Combi PPP Master Mix (TopBio). Further primer details (e.g., original references, fluorescent label employed, concentrations) and PCR cycling parameters are given in Keenan, Bradley, et al. (2013). Amplified fragments were resolved on either a 24 capillary ABI3500xL (University College Cork) or a 96 capillary ABI3730XL (Queens University Belfast) DNA analyzers using POP‐7™ polymer and GeneScan™ 600 LIZ™ dye as size standard (Thermo Fisher Scientific). Genotyping (allele calling) was executed using GeneMarker (SoftGenetics). The genetic sex of each individual was determined based on the presence or absence of an amplified DNA fragment of 108 base pairs at the locus SalmoYF. This fragment is present in male brown trout but not in females.

#### 2.4.3 Tests for genotyping errors

All loci were tested for the presence of genotyping errors due to null alleles or large allele dropout using MICRO‐CHECKER v2.2.3 (Van Oosterhout, Hutchinson, Wills, & Shipley,^2004^). Four loci, BG935488, Ssa85, CAO53293, and MHC‐I showed evidence of high frequencies (8%–12%) of null alleles. Additionally, GENEPOP v4.2 (Rousset, 2008) was used to test each locus in each sampling group for Hardy–Weinberg equilibrium (HWE). *F*_IS_ for these four loci was >0.15 in at least six out of eleven sampling groups, indicating strong heterozygote deficiencies. No other locus exhibited evidence of strong or frequent heterozygote deficiency and, as a result, the four loci identified by MICRO‐CHECKER were excluded from all subsequent analyses. The revised 14 loci dataset was checked for unscored alleles and any individual with fewer than 10 scored loci was removed from downstream analyses (*n* = 9), leaving 844 successfully genotyped samples.

#### 2.4.4 Parentage and sibship analysis

In order to control for the influence of full‐sibling groups on genetic structure analyses and identify mixed‐site half‐sibling groups (i.e., half‐sibs sampled in different places) that could be indicative of nonphilopatric parental breeding behavior, the maximum likelihood method implemented in COLONY v2.0.6.5 (Jones & Wang, 2010) was used to infer parent–offspring relationships as well as half‐ and full‐sibling groupings among all sampled individuals. COLONY input settings were female and male polygamy with inbreeding; dioecious and diploid; medium length run; full likelihood method; updating of allele frequencies; sibship scaling; and no sibship priors. Three replicate runs were conducted with differing seeds, and only assignments that were identified with >90% probability in at least two runs were accepted. Salmonids are not known to display long‐term monogamous breeding behavior over extended spatial scales (Taggart et al., 2001) and, thus, full siblings are likely to be the progeny of matings occurring at a single location. Consequently, full siblings should only become separated geographically through posthatching movement. Half‐sibling families can come about in four ways: (a) a single female mates with two or more males in the same place; (b) a single male mates with two or more females in the same place; (c) a single male moves around and mates with two or more females in different places, and (d) a single female moves around and mates with two or more males in different places. Thus, if scenario (c) and (d) occur regularly, the probability that half siblings hatch in different places should be higher than that for full siblings. The proportion of groups that contained both inflow‐ and outflow‐sampled juvenile members was therefore compared between full and half siblings using the prop.test function in R (with “sites” here corresponding to geographic groups, see below). Groups containing Bunaveela‐sampled members were excluded from this analysis because our sampling indicates the lake is primarily a feeding habitat for older trout that have already moved to the lake from their natal stream. The null hypothesis of equal proportions corresponds to a situation where there are no prehatching differences in spatial distribution of full‐ versus half‐sibling families, or initial differences do exist but are erased by extensive and random posthatching movements. A significant difference in proportions (with inflow–outflow mixed‐site groups more common among half siblings) therefore provides indirect evidence for lack of fidelity to a single spawning stream by polygamous parents, coupled with limited movement of fry or parr between inflow and outflow sites.

In order to avoid bias in population structure analyses that can result from the presence of full‐sibling groups (Anderson & Dunham, 2008; Rodríguez‐Ramilo, Toro, Wang, & Fernández, 2014; Rodríguez‐Ramilo & Wang, 2012), a single individual was selected from each full‐sibling group and retained while all other full siblings were excluded from subsequent analyses. PIT‐tagged individuals that had been detected on antennae were given preference for retention, followed by individuals with the highest number of scored loci, followed by individuals that had been assigned the highest sample identification number.

#### 2.4.5 Calculating population genetic summary statistics

Prior to conducting population structure analyses, temporally distinct samples were merged based on sampling site where genetic differentiation between sampling years was low. To test for temporal genetic structure within our sampled sites, we calculated within‐site between‐time pairwise linearized *F*_ST_ values (Reynolds, Weir, & Cockerham, 1983; Slatkin, 1995) and their significance in Arlequin v3.5.2.2 (Excoffier & Lischer, 2010) using 10,000 dememorization steps and a Markov chain length of 100,000. Without any multiple test correction of p‐values, no within‐site between‐time comparisons were significant, and we thus merged samples across years for all sites, resulting in six geographically defined groups (GGs) (Figure 1; Table 1). Each GG was tested for HWE and linkage disequilibrium (LD; a measure of the independence of loci), using the exact G test in GENEPOP (parameters: 10,000 dememorization steps, 100 batches, and 1,000 iterations per batch). Population summary statistics including observed (*H*_o_) and expected (*H*_E_) heterozygosity, allelic richness (*A*_R_), and inbreeding coefficient (*F*_IS_) were calculated for all GGs using the diveRsity package v1.9.90 (Keenan, McGinnity, Mcginnity, Cross, Crozier, & Prodöhl, 2013) in R (Table 1). Private alleles confined to each GG were identified with the poppr package (Kamvar, Brooks, & Grünwald, 2015; Kamvar, Tabima, & Grünwald, 2014) in R. Effective population size (*N*_e_) was estimated for each GG using the linkage disequilibrium method implemented in NeEstimator V2.1 (Do et al., 2014). This single‐sample method has been shown to provide similar or higher precision in estimates of *N*_e_ than other available methods when applied to highly polymorphic microsatellite data with limited temporal variation such as those used in this study (Waples & Do, 2010).

**Table 1:** Sampling and genetic diversity details of geographic groups (GGs) in the Burrishoole catchment. *N* is the number of samples in each group, *H*_E_ is expected heterozygosity, *H*_O_ is observed heterozygosity_,_ *N*_A_ is the total number of alleles, *A*_R_ is allelic richness, *A*_P_ is number of private alleles (i.e., alleles found only in this sampling group), *F*_IS_ is inbreeding coefficient, *N*_e_ is estimated effective population size based on the linkage disequilibrium method

Sampling site	GG code	Sampling years	*N*	*H*_E_	*H*_O_	*N*_A_	*A*_R_	*A*_P_	*F*_IS_	*N*_e_ (95% CI)
Lower outflow	GG1	2017	27 (27)*	0.72	0.7	120	7.2	21	0.0280 (−0.0587 – 0.1111)	288.90 (98.0‐Infinite)
Upper outflow	GG2	2018	124 (139)*	0.69	0.68	123	6.76	2	0.0141 (−0.0106 – 0.0401)	223.4 (206.8–408.9)
Bunaveela Lough	GG3	2016, 2017, and 2018	465 (497)*	0.69	0.69	140	6.98	13	0.0047 (−0.0086 – 0.0183)	756.7 (591.1–1024.8)
Lower inflow	GG4	2017 and 2018	60 (60)*	0.68	0.68	113	6.57	1	0.0032 (−0.028 – 0.0358)	302.8 (159.7–1695.7)
Middle inflow	GG5	2017 and 2018	65 (81)*	0.7	0.72	118	6.74	0	−0.0284 (−0.0613 – 0.0041)	179.3 (120.7–324.5)
Upper inflow	GG6	2017 and 2018	21 (49)*	0.66	0.68	95	6.01	0	−0.0282 (−0.0908–0.0271)	62.6 (36.7–168.3)

* Numbers enclosed by brackets in the *N* column indicate the number of genotyped samples in each GG prior to removal of full‐sibling samples and samples with <10 scored loci.

#### 2.4.6 Population structure and gene flow

To test for population structure within and among GGs, we used STRUCTURE v.2.3.4 (Falush, Stephens, & Pritchard, 2003, 2007; Hubisz, Falush, Stephens, & Pritchard, 2009; Pritchard, Stephens, & Donnelly, 2000) which quantifies individual admixture and does not require the a priori grouping of individuals by population. STRUCTURE utilizes a Bayesian clustering algorithm to identify the most likely number of distinct genetic clusters (*K*) within a dataset and determines an individual proportional membership (*Q*_i_) for each sample to the inferred cluster(s) of origin so as to minimize departures from HWE within clusters. A hierarchical approach to STRUCTURE analysis was implemented, whereby major genetic groupings within the dataset were identified and separated from one another prior to investigating subtler genetic structure within each of these groupings. Because Bunaveela appears to primarily represent a feeding habitat while the inflow and outflow streams are nursery habitats (see results), a model that utilizes sampling location as a prior for river samples but not for lake samples and allows for admixture was chosen as the most biologically appropriate for our data. A burn‐in period of 100,000 and a Markov chain Monte Carlo (MCMC) of 1,000,000 repetitions after burn‐in were used for each run, and twenty independent iterations with different seed values were conducted for each value of *K* between 1 and 8. Results from all iterations were analyzed with STRUCTURE HARVESTER v0.6.94 (Earl & vonHoldt, 2012) which implements the Evanno method (Evanno, Regnaut, & Goudet, 2005) to indicate the most likely value for *K* based on the rate of change in the log probability of data between successive values of *K*. The “Greedy” algorithm in CLUMPP v1.1.2 (Jakobsson & Rosenberg, 2007) was used to merge the results from each iteration into a combined output file that was used to calculate mean *Q*_i_ values as well as to generate bar plots demonstrating individual membership to each STRUCTURE‐defined cluster.

In order to identify the likely natal stream (i.e., inflow or upper outflow) of mature lake‐feeding trout identified behaviorally as having moved into the inflow only (IO), the outflow only (OO), or both (B), the three behavioral classifications were treated as sampling populations in a STRUCTURE analysis that also included one group comprised of all juveniles sampled in the upper outflow site (GG2) and a second group comprised of all juveniles sampled in the inflow sampling sites (GG4, GG5, and GG6). Location priors were applied to the stream‐sampled groups but not to the IO, OO, or B groups. This analysis was conducted after purely GG‐based analyses had been used to investigate geographic patterns of genetic structure, allowing *K* to be fixed at an appropriate value.

Rates of recent immigration (a proxy for gene flow) between the inflow and the outflow were estimated using BayesAss v3.0.4 (Wilson & Rannala, 2003). BayesAss implements a Bayesian inference framework to estimate the fraction of individuals in a sampled population that are migrants derived from a second (or multiple) sampled population(s) per generation. All samples from the three inflow GGs (GG4, GG5, and GG6) were pooled to form a single “inflow” group (*n* = 146), while the upper outflow samples (GG2) formed a second “upper outflow” group (*n* = 124). All samples used were from trout that were below the threshold maturity FL at time of sampling, with the majority being young‐of‐the‐year. A burn‐in period of 500,000 was used followed by 5,000,000 MCMC iterations with a sampling interval of 1,000 steps. Adjustable acceptance rates fell within the optimal range of 20%–60% suggested by Wilson and Rannala without the need to alter mixing parameters. All analyses were conducted 10 times, each with different initial seed values, and the stationary distributions of the associated chains were compared in order to test for convergence between runs.

Broad spatial patterns of genetic differentiation were assessed within the greater Burrishoole catchment in order to test for evidence of isolation by distance (IBD) and explore broad patterns of gene flow between the upper and lower catchment. Pairwise linearized *F*_ST_ values (Reynolds et al., 1983; Slatkin, 1995) and associated p‐values were calculated among all GGs in Arlequin and the measuring tool in QGIS (v3.8.0‐Zanzibar) was used to measure the minimum traversable waterway distance between all sampling sites. The resulting geographic and genetic distance matrices were assessed for evidence of IBD using a Mantel test (Mantel, 1967) of matrix correspondence as implemented in the “ape” package v5.2 (Paradis & Schliep, 2018) in R.

## 3 RESULTS

### 3.1 Defining maturity status and spawning period

The mean FL of trout that were visually identified as mature during the study period (i.e., ripe, gravid, or spent) was 201.2 mm (*SD* = 36.5 mm, *n* = 322). We thus set 165 mm as the threshold for designating maturity status (~mean – 1 *SD*). At the fish traps, 87.3% of visibly mature trout sampled were above this threshold size. A combination of historical data and recent sampling indicates that outside of the spawning period only 0.46% of trout sampled in the inflow and 0.18% of trout in the upper outflow exceed 165mm, while 26.1% of trout sampled in Bunaveela exceed this length (Appendix Figure A1).

Growth per GDD of recaptured lake‐feeding trout was found to be influenced by both initial FL and *T*_0_. A base temperature of 5°C was selected as the optimum *T*_0_ as model *R* ^2^ values peaked at this base temperature (*R* ^2^ = .70). In order to determine individual maturity status, each tagged trout detected on our antennae during a spawning migration period was thus assigned an estimated fork length for the relevant period using the equation

$$\text{FL2}=\text{FL1}+\left(-0.0303\times \ln\left(\text{FL1}\right)+0.1701\right)\times \text{DD5}$$

where FL2 is fork length at median date of relevant spawning period, FL1 is fork length at date of initial tagging, and DD5 is the sum of growing degree days above a *T*_0_ of 5°C between both dates. Because growth rate as a function of FL1 followed a von Bertalanffy curve, a logarithmic transformation was applied to FL1 (Hordyk, Ono, Sainsbury, Loneragan, & Prince, 2014). The equation above accurately predicts the FL of Bunaveela‐resident trout that were recaptured more than once during the project and whose intervening growth between recapture events had been excluded from the model calibration process (*n* = 10, *R* ^2^ = .91, *p* < .001, RSE = 6.674).

The vast majority (99.0%) of visibly mature trout captured at the Rough River fish trap during the study period were caught between the dates we designated as the spawning period (1 November to 28 February). Similarly, 96.8% of all detections from mature‐sized trout that had been tagged in Bunaveela were recorded on our fluvial antennae between these dates (Appendix Figure A2).

### 3.2 Behavior

A total of 456 trout >70 mm were PIT tagged in Bunaveela during the study period and genetic sex could be confidently determined for 441 of these. Of these, 251 (56.9%) were identified as male and 190 (43.1%) were identified as female. Three hundred and eighty‐four trout were tagged prior to the 2017–2018 spawning period, and 243 of these were estimated to exceed the size threshold for maturity (165 mm) prior to or during that spawning period. The remaining 72 trout were tagged prior to the 2018–2019 spawning period, by which time 450 of the Bunaveela‐tagged trout were estimated to be of mature size. Two hundred and twenty‐nine Bunaveela‐tagged trout were detected on either the inflow antennae, the outflow antennae, or both. Three of these fish were only detected outside of the designated spawning periods and were excluded from subsequent analyses. A further 18 individuals were classified as being below mature length (i.e., estimated FL2 < 165 mm) during the spawning period in which they were detected and were also excluded from analyses, leaving 208 mature‐sized individuals that were detected during one or both spawning periods. Together, these fish produced 112,164 individual detections over the two spawning periods, primarily during the hours of darkness. During the 2017–2018 spawning period, 96.9% and 96.7% of mature trout detections in the inflow and the upper outflow, respectively, were between sunset and sunrise. During the 2018–2019 spawning period, 93.8% and 89.6% of detections in the inflow and the upper outflow, respectively, were between sunset and sunrise. One hundred and sixty‐nine of the 208 mature‐length trout that were detected during a spawning period were detected on the outflow antennae (of which 107 were detected exclusively by the upper outflow antennae, i.e., OO fish), 101 were detected on the inflow antennae (39 exclusively on these antenna, i.e., IO fish), and 62 were detected on both inflow and outflow antennae (B fish) (Table 2). Fifty‐seven and fifty‐eight percent of detected OO and IO fish, respectively, were identified genetically as male, while 64% of B fish were identified as male. The proportion of males within the B group was not found to be significantly different from the proportion of males within the total PIT‐tagged group (57%) (Fischer's exact test: *p* = .18).

**Table 2:** Counts of mature‐length trout detected at the upper outflow antennae and the lower inflow antennae during the 2017–2018 and the 2018–2019 spawning periods

Antennae	Total	Detected at this stream in both spawning periods	Not detected in other stream in either spawning period	Also detected at other stream either spawning period	Detected at other stream in 17–18 spawning period only	Detected at other stream in 18–19 spawning period only
U_OUTFLOW 2017–2018	87	17	46	41	32	5
U_OUTFLOW 2018–2019	99	17	69	30	6	22
U_OUTFLOW Both Years	169	17	107	62	34	24
L_INFLOW 2017–2018	55	5	17	38	30	2
L_INFLOW 2018–2019	51	5	23	28	4	19
L_INFLOW Both Years	101	5	39	62	32	21

During the study period, three out of 3,617 (<0.1%) trout that were tagged in the lower Burrishoole catchment (i.e., downstream of the lower outflow sampling site) were subsequently detected moving past the upper outflow antennae into Bunaveela. All three individuals had been captured and tagged as they moved downstream through the Rough River trap toward the confluence with the most southerly (downstream) section of the outflow (Figure 1). None of these fish were detected by the inflow antennae. None of the 577 trout tagged in the inflow, upper outflow, and Bunaveela (Appendix Table A2) were detected moving downstream past the lower outflow antenna into the lower catchment nor were any of these fish captured as smolts in the fish traps at the tidal limit.

### 3.3 Genetics

#### 3.3.1 Parentage and sibship

COLONY identified 52 full‐sibling groups with *p* ≥ .9 within the total successfully genotyped dataset (*n* = 844) in at least two out of three runs. Full‐sibling groups ranged in size from 2 to 11 individuals. Eighteen groups contained only members sampled in a single stream (inflow or outflow), four groups were dyads containing one inflow and one Bunaveela‐sampled member, one group was a dyad containing one upper outflow and one Bunaveela‐sampled member, and 29 groups were dyads containing two Bunaveela‐sampled members. No full‐sibling groups contained both inflow‐ and outflow‐sampled members. More than 90% of Bunaveela‐sampled dyad members were >85 mm in length (mean FL = 144.3 mm), implying that these Bunaveela‐sampled fish were not young‐of‐the‐year and had most likely moved from their natal stream to the lake, unless some lake‐spawning had occurred (in contrast, mean FL in stream‐sampled groups was 60.1 mm).

Mixed‐site groups were relatively frequent among half‐sibling groups compared with full‐sibling groups, with 74 out of 163 half‐sibling groups (45.4%) composed of two individuals sampled in separate locations (Table 3). While 61 of these mixed‐site half‐sibling dyads contained one member sampled in Bunaveela, 11 dyads contained one member sampled in the inflow and a second member sampled in the outflow. In eight of these 11 dyads, both siblings were sampled as young‐of‐the‐year (<70 mm). When groups containing Bunaveela‐sampled members were excluded from analyses (leaving 44 half‐sibling groups and 18 full‐sibling groups), a significantly higher proportion of half‐sibling groups (25%) contained both an inflow‐ and an outflow‐sampled member compared with full‐sibling groups (0%) (*p* = .049).

**Table 3:** Number of between‐site and within‐site half‐sibling dyads based on maximum likelihood (ML) method in COLONY. Minimum probability required for inclusion of dyad: >90%

	Lower outflow	Upper outflow	Bunaveela	Lower inflow	Middle inflow	Upper inflow
Lower outflow	2					
Upper outflow	2	13				
Bunaveela	7	26	58			
Lower inflow	0	4	7	0		
Middle inflow	2	4	15	2	7	
Upper inflow	0	1	6	1	2	5

Twenty‐four individuals in the total dataset were assigned as parents of other individuals within the dataset (Appendix Table A3). All of these parents were captured within Bunaveela while 12 offspring were sampled in the lake, seven in inflow and five in the upper outflow. Based on the COLONY results, 82 full‐sibling samples, representing ~10% of genotyped individuals, were removed from the dataset prior to performing genetic structure analyses in order to prevent full‐sibling groups from biasing population genetics analyses.

#### 3.3.2 Population genetic summary statistics

After pooling temporal samples within sites, and after sequential Bonferroni correction, locus One‐102‐b showed a significant departure from HWE in the lower outflow group (GG1). No other locus departed significantly from HWE in any other GG, and no locus pairs showed significant LD in any GG. Genetic structure analyses conducted both with and without the One‐102‐b locus were highly congruent, indicating that this single departure from HWE had no significant impact on observed patterns of genetic structure between GGs. Consequently, results described hereafter refer to analyses utilizing 14 loci, include locus One‐102‐b.

#### 3.3.3 Population structure and gene flow

**Figure 2:**
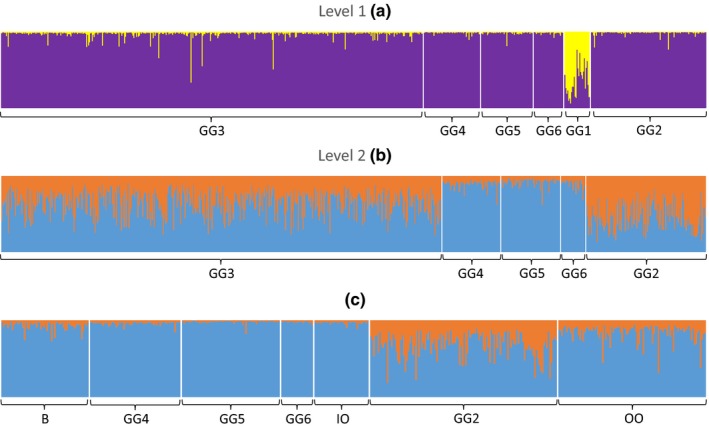
STRUCTURE plot of (a) all six GGs (*K* = 2), (b) five upper catchment GGs (GG2–GG6) (*K* = 2), and (c) pooled inflow GGs (GG4, GG5, and GG6), upper outflow (GG2), and the three behaviorally defined lake‐sampled groups (B = Individuals detected moving into both the inflow and the outflow; IO = Individuals detected moving into the inflow only; OO = Individuals detected moving into the outflow only) (*K* = 2)

**Figure A3:**
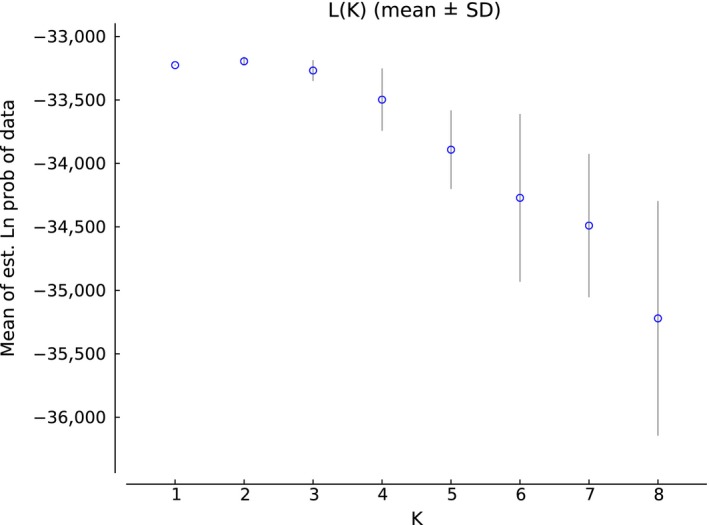
Mean Ln probability (STRUCTURE method) of *K* = 1–8. All 6 GGs

**Figure A4:**
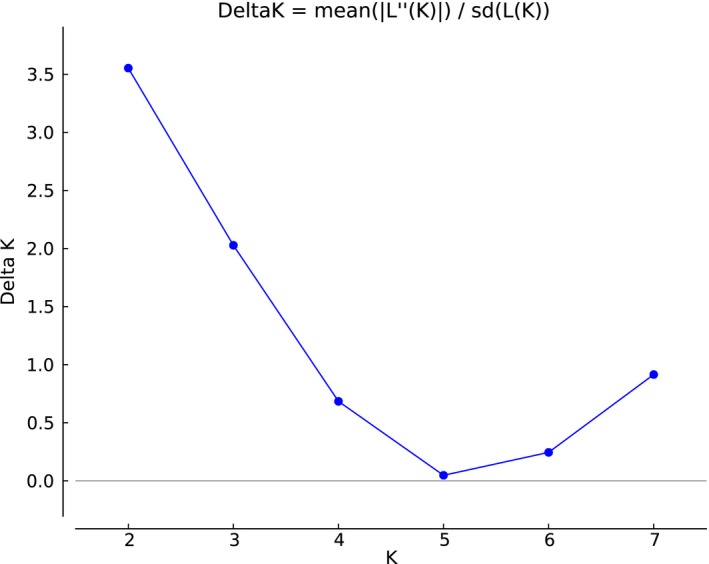
Delta *K* (Evanno method) of *K* = 2–7. All 6 GGs

Level one of hierarchical STRUCTURE analysis identified evidence of two genetic clusters (*K* = 2) (Appendix Figures A3 and A4). These two clusters could be broadly described as “upper catchment” and “lower outflow.” The upper catchment cluster was predominantly composed of individuals from the five uppermost sampling sites (GG2–GG6), and individuals from these GGs had a mean assignment score (*Q*_i_) to this cluster of 0.97 (*SD* = 0.06). The lower outflow cluster was predominantly composed of individuals from the lower outflow (GG1), with lower and more variable individual assignment scores (mean *Q*_i_ to the lower catchment cluster was 0.65 ± 0.19) (Figure 2a).

**Figure A5:**
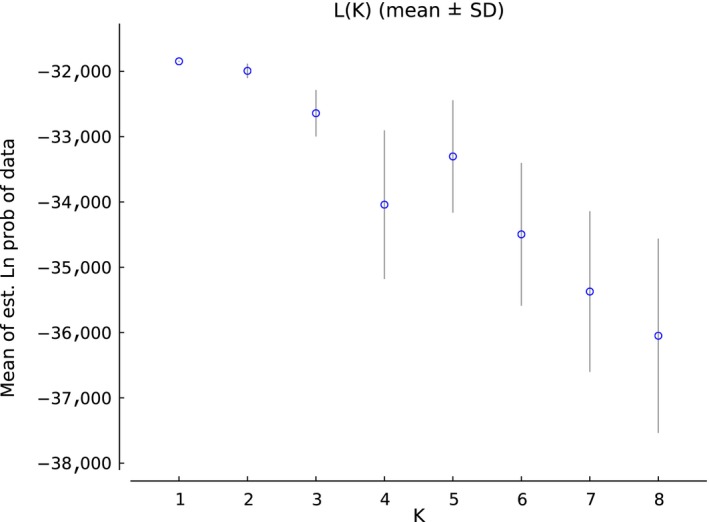
Mean Ln probability (STRUCTURE method) of *K* = 1–8. GG2–GG6

**Figure A7:**
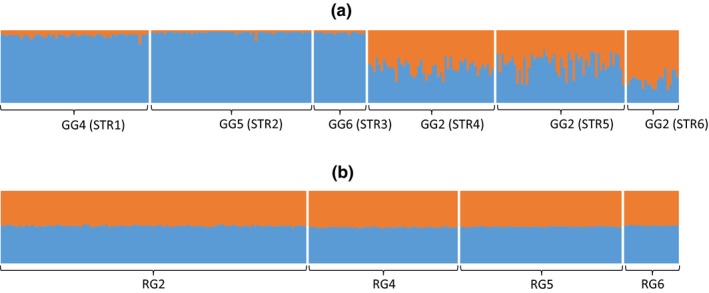
STRUCTURE plots of inflow (GG4–GG6) and upper outflow (GG2) geographic groups (*K* = 2), in which: (a) GG2 was divided into three population groups (STR4, STR5, and STR6) equal in size to GG3, GG4, and GG5 prior to analysis. (b) All samples from the inflow and upper outflow groups (GG2, GG4, GG5, and GG6) were randomly assigned among four randomized groups (RGs) equal in size to GG2, GG4, GG5, and GG6 prior to analysis

In order to investigate lower hierarchical levels of genetic structure within the upper catchment cluster, a STRUCTURE analysis was performed on the five GGs associated with the upper catchment cluster (GG2–GG6). Although the mean ln(*K*) values for this analysis were marginally higher for *K* = 1 than *K* = 2, this difference was minor when compared with all other modeled values of *K*, suggesting that ln(*K*) plateaus at *K* = 2 (Appendix Figure A5). Furthermore, at *K* = 2, a large proportion of individuals had strong assignment to one cluster or the other, and with spatial nonhomogeneity in the distribution of assignment scores (Figure 2b), a pattern consistent with the presence of genuine population structure (Pritchard, Wen, & Falush, 2007). At *K* = 2, most individuals in the three inflow groups (GG4, GG5, and GG6) assign strongly to a single “inflow” cluster with low levels of admixture (mean *Q*_i_ = 0.90, *SD* = 0.06). In contrast, the Bunaveela and upper outflow (GG3 and GG2) show evidence of significant admixture, with mean inflow cluster *Q*_i_ values of 0.64 (*SD* = 0.16) and 0.45 (*SD* = 0.17) respectively. These assignment patterns were maintained throughout further STRUCTURE runs in which GG2 (upper outflow) was split into three groups of equal size to the three inflow GGs (Appendix Figure Figure A7a), but such patterns disappeared when individual samples from the inflow and upper outflow GGs were randomly assigned among four predefined groups matched in size to the four donor GGs (Appendix Figure A7b). These findings indicate that the patterns shown in Figure 2 represent genuine geographically based structure rather than artifacts from Bayesian priors. No evidence of lower hierarchical levels of genetic substructuring was detected within the inflow or outflow GGs during subsequent analyses of these groups.

When the OO, IO, and B groups of mature PIT‐tagged lake‐sampled fish were included in a STRUCTURE analysis (fixed at *K* = 2) that also included an upper outflow and a merged inflow group (with the latter two groups composed offry and parr sampled in the streams), each of the three behaviorally defined groups exhibited distinct assignment patterns that resemble the characteristic assignment patterns of the inflow and upper outflow groups. In particular, the IO group, the B group, and the merged inflow group were characterized by consistently high individual assignment to a single “inflow” cluster (Figure 2c) with mean *Q*_i_ values to this cluster of 0.97 (*SD* = 0.02), 0.90 (*SD* = 0.06), and 0.97 (*SD* = 0.02), respectively. In contrast, the OO group and the outflow‐sampled juvenile group were both characterized by higher and relatively variable assignment to a second cluster, indicative of greater admixture within these groups (mean *Q*_i_ to the second cluster for these groups is 0.17 (*SD* = 0.11) and 0.33 (*SD* = 0.16), respectively).

BayesAss results indicate that recent migration rates between the inflow and upper outflow streams have been strongly asymmetrical and are characterized by a predominantly downstream direction of migration from the inflow to the outflow. Less than 1% (0.95%) of the trout in the inflow are estimated to be migrants derived from the outflow group (per generation), while 32.02% of trout in the upper outflow are estimated to be migrants derived from the inflow group (per generation). The associated 95% credible intervals, a Bayesian analogue to confidence intervals, are 0% to 2.4% and 30% to 34.01%, respectively. There was a high degree of convergence between multiple runs (Appendix Table A4), indicating that that the asymmetric pattern detected by BayesAss reflects genuine differences in migration rates between the inflow and outflow. Genetic diversity generally increased from upstream to downstream, with the minimum *A*_R_ recorded in the uppermost group (6.01 in the upper inflow, GG6), and the maximum *A*_R_ recorded in the lowest group (7.02 in the lower outflow, GG1).

**Figure 3:**
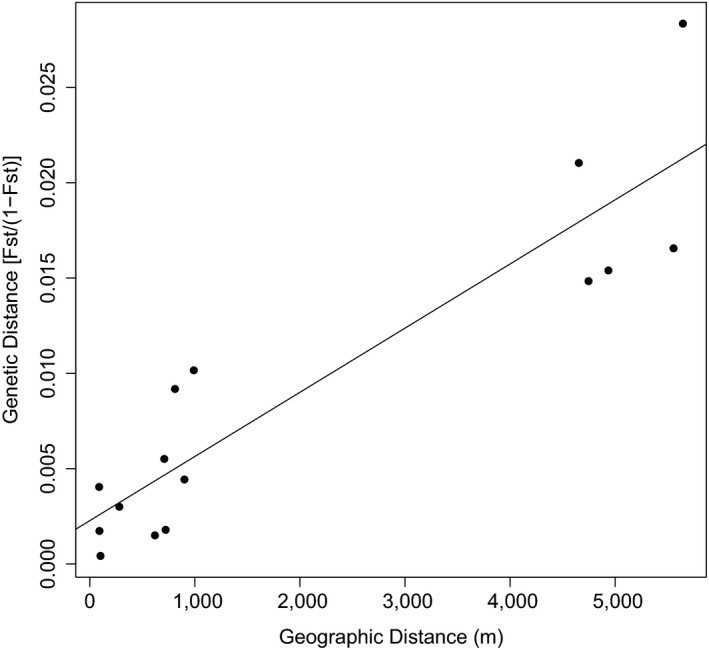
Relationship between genetic distance [*F*_ST_/(1 − *F*_ST_)] and geographic distance (m) for all geographic group (GG) pairings. *R* ^2^ = 0.83, *p* < .001

Isolation‐by‐distance analyses revealed that genetic distance between GG pairs, expressed as Slatkin's pairwise linearized *F*_ST_ [*F*_ST_/(1 − *F*_ST_)], ranged from <0.001 to 0.028 while geographic distance between sampling sites ranged from 89 to 5,645 m (Table 4). Mantel test results indicate the occurrence of a positive correlation between genetic distance and geographic distance matrices (Z = 525.47, *p* = .013), a pattern of genetic structure consistent with isolation by distance (see Figure 3). All of the highest pairwise *F*_ST_ values are associated with GG pairs that include the lower outflow group, GG1 (*F*_ST_ = 0.015–0.029), indicating that the trout sampled at this site belong to the most genetically distinct GG. The lower outflow site is also the most geographically distant site from Bunaveela, located 4747m downstream from the lake. When GG1 was excluded from analysis, the Mantel test revealed evidence of significant IBD within the five upper catchment groups, GG2–GG6 (*z*‐statistic = 25.82, *p* = .033). The three inflow groups (GG4–GG6) share nonsignificant pairwise *F*_ST_ values of <0.006, indicating high rates of gene flow among all GGs within the inflowing stream.

**Table 4:** Pairwise genetic distances and geographic distances between all geographic groups (GGs). Genetic distances [*F*_ST_/(1 − *F*_ST_)] are located in the bottom left diagonal. Geographic distances (m) are located in the top right diagonal. Genetic distances with significant associated *p*‐values (after applying as sequential Bonferroni correction) are indicated by bold text

	Lower outflow	Upper outflow	Bunaveela	Lower inflow	Middle inflow	Upper inflow
Lower outflow	0	4,655	4,747	4,936	5,556	5,645
Upper outflow	**0.02104**	0	92	281	901	990
Bunaveela	**0.01484**	0.00173	0	102	722	811
Lower inflow	**0.0154**	0.003	0.00042	0	620	709
Middle inflow	**0.01656**	**0.00443**	0.00179	−0.00166	0	89
Upper inflow	**0.02835**	**0.01016**	**0.00918**	0.00551	0.00404	0

## 4 DISCUSSION

Previous studies of brown trout have shown that populations spawning in inflowing versus outflowing streams of the same lake are often genetically and phenotypically differentiated, implying limited gene flow among them and potential local adaptation (Ferguson & Taggart, 1991; Jonsson et al., 1994; Linløkken, Johansen, & Wilson, 2014; Massa‐Gallucci et al., 2010; Palmé et al., 2013). In cases where no obvious physical barriers to dispersal exist, some combination of prezygotic behavioral isolating mechanisms (that reduce the likelihood of inflow‐origin fish straying into outflow streams and successfully mating there, or vice versa) and postzygotic ecological barriers (reduced fitness of hybrid offspring) may constrain effective dispersal and thus gene flow. Here, we explored these issues at a microgeographic scale, that is, a fine spatial scale that is within the typical “dispersal neighborhood” of the species (Richardson et al., 2014). Our telemetry results showed that the potential for dispersal between inflow and outflow streams is high in the Bunaveela system, given that ~30% of lake‐tagged trout that were detected by our antennae were detected moving into both streams. Genetic sibship analysis indicated that some fry sampled in nursery habitat in the inflow stream had half siblings present in the outflow stream (and vice versa), suggesting that one of their parents had reproduced successfully in both streams. This indirect evidence for gene flow was consistent with the rather weak genetic differentiation we documented between the streams. Importantly, however, the genetic data also revealed higher rates of recent migration from the inflow into the outflow than vice versa. Given the short dispersal distances involved and the lack of any obvious physical barriers, our findings point collectively toward interesting asymmetries in pre‐ or postzygotic isolating mechanisms, which we discuss further below.

### 4.1 Interpreting the telemetry and population structure results in light of each other

A key goal of this study was to couple PIT telemetry with microsatellite‐based population genetic inference to obtain complementary insights into homing/straying behaviors and the implications for population structuring. A priori, we expected to find some genetic differentiation between the inflow and outflow streams, given that (a) brown trout can exhibit genetic structuring at fine spatial scales (Carlsson et al., 1999; Lobón‐Cerviá & Sanz, 2017), (b) previous studies (see above) have found genetic differences between inflow and outflow streams, and (c) isolation‐by‐adaptation mechanisms can be reasonably hypothesized (see below). We indeed documented subtle population structuring within the upper Burrishoole catchment, with two putative clusters that corresponded to some degree, but not perfectly, with inflow versus outflow spawning streams. The weak nature of this structure precluded us from being able to pinpoint cases of “pure straying” behavior, which would require assigning lake‐tagged individuals with high confidence to a given natal stream (inflow or outflow) and subsequently demonstrating with PIT telemetry that they were only detected in the opposite stream during the spawning season. However, the existence of “pure homing” and mixed homing/straying tactics could be tentatively inferred. Lake‐tagged fish detected during the spawning period in the inflow stream only (IO group) exhibited consistently high individual assignment (mean *Q*_i_ = 0.97, *SD* = 0.02) to a single putative “inflow cluster”; thus, the IO group, which comprised ~39% of all fish detected on the inflow antennas, likely represented mostly “inflow‐origin homers.”

A second genetic cluster corresponded most closely with the upper outflow (GG2), as indicated by the fact that the mean cluster 2 *Q*_i_ for this group was 0.55 (*SD* = 0.17) in the STRUCTURE analysis that excluded the behavioral groups (Figure 3b). The lower and more variable assignment here compared to the very high and consistent assignment of inflow groups (GG4–GG6) to the putative inflow cluster (cluster 1) likely reflects substantial net immigration from inflow to outflow; thus, there is weak genetic differentiation between them and high levels of admixture in the outflow. Additional sampling further downstream in the outflow would perhaps have revealed increasing assignment to cluster 2, making the overall distinction with respect to the inflow cluster clearer. When the behavioral groups were included in the analysis, OO fish importantly exhibited higher, although again more variable, assignments to the putative outflow cluster (mean cluster 2 *Q*_i_ = 0.17, *SD* = 0.11) compared with IO fish (mean cluster 2 *Q*_i_ = 0.03, *SD* = 0.02) or B fish (mean cluster 2 *Q*_i_ = 0.10, *SD* = 0.06). This again indicates admixture in the OO group and suggests that these fish were of mixed ancestry, consistent with gene flow from the inflow to the outflow. Thus, OO fish may represent “outflow‐origin homers” (born in outflow, returned to outflow), “inflow‐origin strayers” (born in inflow, moved to outflow), or some mix of both. The genetic assignment patterns point more toward OO fish being predominantly outflow‐origin homers, but we have less confidence in this inference relative to our inference regarding the IO fish. Interestingly, the B group were characterized by individual assignment scores to the putative inflow cluster (mean *Q*_i_ = 0.90, *SD* = 0.06) that were lower and more variable relative to IO fish assignments to the same cluster, but higher and less variable relative to OO fish. This suggests that many of the B group fish originated in the inflow stream—having a genetic signature that was more “inflow in nature”—but may have actually spawned in both streams, again consistent with net gene flow from inflow to outflow. Thus, B fish may be both homers and strayers, although clearly the definition of these terms is contingent on the criteria one uses to define “distinct” genetic populations, which may not map cleanly onto population units defined using demographic criteria (Waples & Gaggiotti, 2006).

The identification of juvenile half‐sibling dyads (i.e., sharing a single parent) containing both inflow‐ and outflow‐sampled fry indicates that some individuals indeed spawn in both streams. An alternative scenario whereby half siblings were all born in the same stream, but some then moved as fry to the other stream seems highly unlikely, as these fry were sampled early in life on the nursery grounds prior to when extensive dispersion is believed to occur. Additionally, the proportion of dyads containing both inflow‐ and outflow‐sampled members relative to same site dyads was significantly higher among half siblings than among full siblings, indicating that juvenile movement between sites (which is likely to be equally common within half‐ and full‐sibling groups) does not account for the prevalence of these inflow–outflow half‐sibling dyads.

### 4.2 Asymmetric dispersal and the maintenance of genetic structure between inflow and outflow

Bayesian analyses indicated that contemporary migration rates between the two streams are strongly asymmetrical in a predominantly downstream direction, implying that inflow‐to‐outflow dispersal significantly exceeds outflow‐to‐inflow dispersal. The program BayesAss estimated that in recent years, ~31% of trout in the upper outflow are migrants derived from the inflow (strayers), which accorded well with our telemetry observation that ~37% of ostensible outflow spawners belonged to our B group, that is, they were detected during the spawning period in both streams. These findings were also consistent with the Bayesian clustering (STRUCTURE) results, whereby juvenile trout sampled in the inflow were characterized by consistently high individual assignment to a single cluster, indicating that effective migrants into this stream are relatively rare. In contrast, fry sampled in the outflow exhibited higher and more variable assignment to a second cluster, indicating that comparatively high rates of immigration result in greater admixture at this location. Taking all genetics and telemetry results together, it appears that trout originating in the inflow are more prone to between‐stream dispersal than trout originating in the outflow.

Flow direction effects may combine with behavioral and olfactory mechanisms to bias dispersal in favor of inflow to outflow. For example, inflow‐born fry may be easily washed downstream into the lake before olfactory imprinting on inflow water, while outflow fry cannot be washed into the lake—they must actively swim against the flow to reach the lake. Additionally, as spawning time approaches, lake‐dwelling outflow‐origin fish that approach the mouth of the inflow would receive odor cues that “smell wrong,” assuming they first imprinted on outflow water before moving to the lake. In contrast, lake‐dwelling inflow‐origin fish would presumably detect no odor cues from the outflow stream until they enter it, so such exploratory movements may be more common among inflow‐origin trout. Moreover, for spawning‐age fish of either origin, moving from the lake into the outflow can be completely passive whereas moving into the inflow requires active locomotion.

More generally, asymmetry in gene flow tends to be associated with systems driven by directional physical processes such as flowing water (Sundqvist, Keenan, Zackrisson, Prodöhl, & Kleinhans, 2016), wind (Cook & Crisp, 2005; Sanmartı et al., 2007), and pelagic currents (Pringle et al., 2011; Storch & Pringle, 2018). In such scenarios, prevailing air or water currents tend to act as prezygotic mechanisms that promote dispersal from “upstream” populations to “downstream” populations while restricting dispersal in the opposite direction. This asymmetric dispersal coupled with differences in selective pressures (and possible variation in habitat quality) can lead to interesting source‐sink population dynamics with complex evolutionary outcomes (Kawecki & Holt, 2002). Additionally, net dispersal from upstream to downstream should lead to higher genetic diversity in downstream populations relative to upstream populations, all else being equal. Indeed, the most downstream group within our set of samples (lower outflow, GG1) exhibited the highest genetic diversity (Table 1) while the uppermost group (upper inflow, GG6) exhibited the lowest genetic diversity. This broad pattern of decreasing genetic diversity from downstream to upstream suggests that downstream‐biased gene flow operates within the Burrishoole catchment at various scales. It is possible that two small waterfalls, the first located upstream of the lower outflow (GG1) and second located just downstream of the upper inflow (GG6), serve to limit upstream gene flow despite being navigable by trout traveling in either direction.

Given putative net gene flow from inflow to outflow, why does the upper outflow not become genetically indistinguishable from the inflow spawning stream? One possibility is that the upper outflow sections receive migrants from genetically distinct subpopulations lower down in the Burrishoole system, but the inflow stream does not, contributing to the maintenance of genetic differences between them. Indeed, a small number of trout that were PIT‐tagged in the lower catchment were subsequently detected in the upper outflow, but never in the inflow stream. The genetic contribution of such putative upstream strayers, in conjunction with that provided by downstream strayers from the inflow, may thus account for the pattern of high admixture observed among trout sampled in the upper outflow, a pattern that is largely absent from the inflow samples.

An alternative explanation for the maintenance of inflow–outflow genetic differentiation is that reduced fitness of hybrids resulting from matings between inflow‐ and outflow‐origin fish acts as a postzygotic isolating barrier to effective dispersal. Inflow‐born fry must move downstream to reach productive lake‐rearing habitat, whereas outflow‐born fry must move upstream to reach the lake. A genetic basis for fry movement direction has previously been shown in inflow versus outflow systems (Jonsson et al., 1994), and thus, alleles for downstream fry movement may be selected against in outflow‐born hybrids and vice versa in inflow‐born hybrids. F1 hybrids may therefore die at higher rates or grow less well due to not reaching lake habitat, in each stream, effectively reducing rates of gene flow between the streams. Such isolation‐by‐adaptation processes can also contribute to genetic differentiation at neutral markers, for which divergence is promoted by genetic drift and constrained by gene flow (Nosil et al., 2008; Orsini et al., 2013). The low to moderate *N*_e_ values (Table 1) estimated for these populations imply that nontrivial genetic drift may indeed be in operation. The existence of postzygotic barriers could also foster the evolution of prezygotic isolating mechanisms, that is, reinforcement, where, for example, inflow‐origin fish “prefer” to mate with other inflow‐origin fish and vice versa in order to avoid producing less‐fit hybrids. It is also worth noting that while the isolation‐by‐distance pattern we document (Figure 3) at a broader catchment scale is consistent with simple isolation‐by‐dispersal limitation and asymmetric gene flow, it could also be produced by isolation‐by‐adaptation process if ecological dissimilarity—and therefore the extent of local adaptation—also increases with distance (Orsini et al., 2013).

While our research strongly suggests that mature trout that move from Bunaveela to both the inflow and the outflow often breed successfully in both streams, the nocturnal nature of these activities prevented us from directly observing individuals during spawning. Consequently, we cannot unequivocally confirm that the putative straying behavior detected during this study resulted in effective dispersal. On the other hand, it is possible for female salmonids to shed PIT tags during spawning (Bateman, Gresswell, & Berger, 2009; Foldvik & Kvingedal, 2018; Taylor et al., 2011). Indeed, such tag loss by females may account for the male bias observed among putative strayers. If so, the actual proportion of strayers among the trout tagged during this study may exceed the proportion reported here. Finally, although brown trout primarily spawn in fluvial habitats, they are capable of successfully spawning in lakes if suitable hydrological conditions are present (Arostegui & Quinn, 2019). It is possible that some of the mature‐sized lake‐tagged trout that were not detected in either the inflow or outflow were in fact lacustrine spawners. Predation by large brown trout, otters (*Lutra lutra*), eels, cormorants (*Phalacrocorax carbo*), and herons (*Ardea cinerea*) may also account for some of the discrepancy between the number of trout that we tagged and the number we subsequently detected. Due to the difficulty of operating efficient PIT antennae in lakes, and the difficulty of distinguishing lake feeding from lake‐spawning related detections, it was not possible to assess the local prevalence of lake‐spawning during this project, nor the influence of such behavior on local population structure.

### 4.3 Concluding remarks

The geographic scale at which natal philopatry and natal dispersal operate plays an important role in regulating gene flow and determining population structure within the landscape. Our research here indicates that a small expanse of intervening lake habitat can have a significant influence on rates of dispersal and philopatry among trout populations that spawn in inflowing and outflowing streams. Furthermore, it appears that in such scenarios inflow‐to‐outflow dispersal may significantly exceed outflow‐to‐inflow dispersal. Analogous asymmetric dispersal patterns are found in various species, with important demographic (Storch & Pringle, 2018) and evolutionary (Kawecki & Holt, 2002) consequences. Isolation‐by‐adaptation type mechanisms may promote the maintenance of genetic differentiation at neutral, in addition to non‐neutral, loci between inflow and outflow populations, while asymmetric dispersal will tend to increase genetic diversity of outflow populations. Through these processes, the presence of lake habitat between inflow and outflow streams, despite providing no physical barrier to movement or dispersal, may facilitate the evolution and persistence of local adaptations in salmonid populations at finer geographic scales than has been traditionally suggested in the literature (Adkison, 1995; Fraser et al., 2011). Restocking programs that fail to adequately consider the geographic scale of local adaptations or the implications of asymmetrical gene flow between captive and wild populations (Baskett & Waples, 2012) may result in suboptimal performance of stocked fish and potentially threaten the long‐term performance of extant local populations by diluting locally adapted traits (McGinnity et al., 2007, 2009; Mobley et al., 2019).

## CONFLICT OF INTEREST

The authors declare no conflict of interest.

## AUTHOR CONTRIBUTIONS

RF, TR, PMcG, and RP conceived and designed the experiment. RF and RP collected the data. RF, TR, JC, KP, and PP analyzed the data. RF led the writing of the manuscript. DC ensured compliance with scientific animal protection. All authors contributed to drafts of the manuscript and gave final approval for publication.

## Figures and Tables

**Figure A6:**
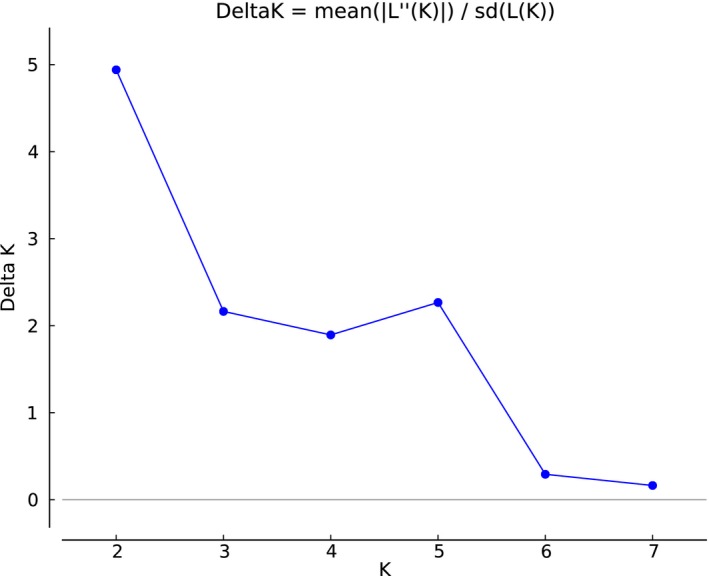
Delta *K* (Evanno method) of *K* = 2–7. GG2–GG6

## Data Availability

Sampling data, genetic data, PIT telemetry detection data, and growth data are available via the Marine Institute Ireland Data Catalogue, https://doi.org/10.20393/e9395f08-67cb-422a-9ed6-dc16ad5613c8.

## APPENDIX A xxxx

**Table A1:** Hydrological properties of Bunaveela Lough, the upper outflow and the inflow streams

Site	pH	Conductivity (µS/cm)	Alkalinity (CaCO_3_ equivalent mg/l)	Nitrogen (mg/l)	Phosphorous (μg/l)
Bunaveela	7.2	86	33.0	0.657	17.66
Inflow	7.23	116	30.87	0.274	15
Upper outflow	7.05	96.65	10.14	0.238	7

**Table A2:** Summary of trout, salmon and char that were caught, tagged or recaptured within the Burrishoole catchment during the duration of the project

Date	Site	Number of seine net hauls or electrofishing passes	Trout tagged FL > 70 mm	Trout FL < 70 mm	Trout recaptured (previously tagged)	Salmon tagged FL > 70 mm	Salmon FL < 70 mm	Salmon recaptures (previously tagged)
11/10/2016	Bunaveela	5	44	0	0	10	0	0
12/10/2016	Lodge stream	3	10	0	0	7	0	0
21/06/2017	Bunaveela	5	147	13	1	29	5	0
23/06/2017	Feeagh	5	71	0	4	80	1	4
28/06/2017	Glenamong River	3	17	0	0	112	0	0
29/06/2017	Lower outflow	3	28	1	0	139	7	0
16/08/2017	Bunaveela	6	102	16	14	20	2	1
21/08/2017	Feeagh	6	52	0	17	28	3	21
06/09/2017	Lower inflow	3	20	3	0	8	0	0
06/09/2017	Middle inflow	3	16	8	0	12	1	0
06/09/2017	Upper inflow	3	13	10	0	10	6	0
26/10/2017	Bunaveela	6	91	6	19	10	5	1
27/10/2017	Feeagh	6	31	0	12	11	0	9
15/08/2018	Upper outflow	3	23	129	0	0	0	0
05/09/2018	Lower inflow	3	28	24	0	0	0	0
05/09/2018	Middle inflow	3	6	53	0	14	0	0
05/09/2018	Upper inflow	3	15	12	0	0	0	0
24/10/2018	Bunaveela	4	41	6	10	4	3	0
08/11/2018	Bunaveela	3	31	3	10	2	0	0
27 Dates	Rough River	3	1,227	326	315	1,111	3,539	270
Daily	Rough River traps	NA	1759	165	287	546	878	192
Daily	Tidal limit traps	NA	450	NA	41	NA	NA	357
	Total	NA	4,223	774	730	2,153	4,450	855

**Table A3:** Parentage assignment for offspring from each GG. All parents assigned to offspring within the total dataset came from the Bunaveela GG, including parents of fry sampled in the inflow and outflow streams

Offspring site	Bunaveela	Lower inflow	Middle inflow	Upper inflow	Lower outflow	Upper outflow	Total
Number assigned to a Bunaveela‐sampled parent	12	4	3	0	0	5	24

**Table A4:** BayesAss migration estimates between the Inflow and Upper Outflow. 10 runs conducted with different starting seeds in order to assess convergence between runs. m[a][b] describes proportion of immigrants into population [a] derived from population [b] per generation

	m[UpperOutflow][Inflow]	m[UpperOutflow][UpperOutflow]	m[Inflow][Inflow]	m[Inflow][UpperOutflow]
Run1	0.3196	0.6804	0.9907	0.0093
Run2	0.3194	0.6806	0.9901	0.0099
Run3	0.3198	0.6802	0.9902	0.0098
Run4	0.3205	0.6795	0.9904	0.0096
Run5	0.3197	0.6803	0.9907	0.0093
Run6	0.3199	0.6801	0.9905	0.0095
Run7	0.3203	0.6797	0.9909	0.0091
Run8	0.3196	0.6804	0.9903	0.0097
Run9	0.3197	0.6803	0.9905	0.0095
Run10	0.32	0.68	0.9907	0.0093
Mean	0.31985	0.68015	0.9905	0.0095
StDev	0.000320156	0.000320156	0.000240832	0.000240832

## References

[ece35937-bib-0001] Adkison, M. D. (1995). Population differentiation in Pacific salmon: Local adaptation, genetic drift, or the environment? Canadian Journal of Fisheries and Aquatic Sciences, 52, 2762–2777. 10.1139/f95-865

[ece35937-bib-0002] Anderson, E. C. , & Dunham, K. K. (2008). The influence of family groups on inferences made with the program Structure. Molecular Ecology Resources, 8(6), 1219–1229. 10.1111/j.1755-0998.2008.02355.x 21586009

[ece35937-bib-0003] Arostegui, M. C. , & Quinn, T. P. (2019). Reliance on lakes by salmon, trout and charr (Oncorhynchus, Salmo and Salvelinus): An evaluation of spawning habitats, rearing strategies and trophic polymorphisms. Fish and Fisheries, 20(4), 775–794.

[ece35937-bib-0004] Baskett, M. L. , & Waples, R. S. (2012). Evaluating alternative strategies for minimizing unintended fitness consequences of cultured individuals on wild populations. Conservation Biology, 27(1), 83–94. 10.1111/j.1523-1739.2012.01949.x 23082984

[ece35937-bib-0005] Bateman, D. S. , Gresswell, R. E. , & Berger, A. M. (2009). Passive integrated transponder tag retention rates in headwater populations of coastal cutthroat trout passive integrated transponder tag retention rates in headwater populations of coastal cutthroat trout. North American Journal of Fisheries Management, 29, 653–657. 10.1577/M07-169.1

[ece35937-bib-0006] Boltaña, S. , Sanhueza, N. , Aguilar, A. , Gallardo‐Escarate, C. , Arriagada, G. , Valdes, J. A. , … Quiñones, R. A. (2017). Influences of thermal environment on fish growth. Ecology and Evolution, 7, 6814–6825. 10.1002/ece3.3239 28904762 PMC5587470

[ece35937-bib-0007] Bowler, B. (1975). Factors influencing genetic control in lakeward migrations of cutthroat trout fry. Transactions of the American Fisheries Society, 3, 474–482. 10.1577/1548-8659(1975)104<474:FIGCIL>2.0.CO;2

[ece35937-bib-0008] Bradbury, I. R. , Hamilton, L. C. , Robertson, M. J. , Bourgeois, C. E. , Mansour, A. , & Dempson, J. B. (2013). Landscape structure and climatic variation determine Atlantic salmon genetic connectivity in the Northwest Atlantic. Canadian Journal of Fisheries and Aquatic Sciences, 71(2), 246–258. 10.1139/cjfas-2013-0240

[ece35937-bib-0009] Brannon, E. L. (1972). Mechanisms controlling migration of sockeye salmon fry. Canada: New Westminster.

[ece35937-bib-0010] Carlsson, J. , Olsén, K. H. , Nilsson, J. , Øverli, Ø. , & Stabell, O. B. (1999). Microsatellites reveal fine‐scale genetic structure in stream‐living brown trout. Journal of Fish Biology, 55(6), 1290–1303. 10.1111/j.1095-8649.1999.tb02076.x

[ece35937-bib-0011] Chezik, K. A. , Lester, N. P. , & Venturelli, P. A. (2014). Fish growth and degree‐days II: Selecting a base temperature for an among‐population study. Canadian Journal of Fisheries and Aquatic Sciences, 71(9), 1303–1311. 10.1139/cjfas-2013-0615

[ece35937-bib-0012] Cook, L. G. , & Crisp, M. D. (2005). Directional asymmetry of long‐distance dispersal and colonization could mislead reconstructions of biogeography. Journal of Biogeography, 32, 741–754. 10.1111/j.1365-2699.2005.01261.x

[ece35937-bib-0013] de Eyto, E. , Dillane, M. , Cooney, J. , Hughes, P. , Murphy, M. , Nixon, P. , … Rouen, M. (2019). Water quality and meteorological data from the Lough Feeagh Automatic Water Quality Monitoring Station (AWQMS), 2004–2017 Marine Institute, Ireland. Marine Institute Ireland, Retrieved from http://data.marine.ie/data/edd58462-ae36-44b2-bf36-0ef06c6e8357.zip

[ece35937-bib-0014] de Eyto, E. , McGinnity, P. , Huisman, J. , Coughlan, J. , Consuegra, S. , Farrell, K. , … Stet, R. J. M. (2011). Varying disease‐mediated selection at different life‐history stages of Atlantic salmon in fresh water. Evolutionary Applications, 4, 749–762. 10.1111/j.1752-4571.2011.00197.x 25568020 PMC3352546

[ece35937-bib-0015] De Fraipont, M. , Clobert, J. , John Alder, H. , & Meylan, S. (2000). Increased pre‐natal maternal corticosterone promotes philopatry of offspring in common lizards *Lacerta vivipara* . Journal of Animal Ecology, 69, 404–413. 10.1046/j.1365-2656.2000.00405.x

[ece35937-bib-0016] Dillane, E. , McGinnity, P. , Coughlan, J. P. , Cross, M. C. , De Eyto, E. , Kenchington, E. , … Cross, T. F. (2008). Demographics and landscape features determine intrariver population structure in Atlantic salmon (*Salmo salar* L.): The case of the River Moy in Ireland. Molecular Ecology, 17(22), 4786–4800. 10.1111/j.1365-294x.2008.03939.x 19140972

[ece35937-bib-0017] Dingle, H. , & Drake, V. A. (2007). What is migration? BioScience, 57(2), 113–121. 10.1641/B570206

[ece35937-bib-0018] Dionne, M. , Caron, F. , Dodson, J. J. , & Bernatchez, L. (2008). Landscape genetics and hierarchical genetic structure in Atlantic salmon: The interaction of gene flow and local adaptation. Molecular Ecology, 17(10), 2382–2396. 10.1111/j.1365-294X.2008.03771.x 18430145

[ece35937-bib-0019] Dittman, A. H. , & Quinn, T. P. (1996). Homing in Pacific salmon: Mechanisms and ecological basis. The Journal of Experimental Biology, 199(1), 83–91. 9317381 10.1242/jeb.199.1.83

[ece35937-bib-0020] Do, C. , Waples, R. S. , Peel, D. , Macbeth, G. M. , Tillett, B. J. , & Ovenden, J. R. (2014). NeEstimator v2: Re‐implementation of software for the estimation of contemporary effective population size (Ne) from genetic data. Molecular Ecology Resources, 14(1), 209–214. 23992227 10.1111/1755-0998.12157

[ece35937-bib-0021] Earl, D. A. , & vonHoldt, B. M. (2012). STRUCTURE HARVESTER: A website and program for visualizing STRUCTURE output and implementing the Evanno method. Conservation Genetics Resources, 4(2), 359–361. 10.1007/s12686-011-9548-7

[ece35937-bib-0022] Evanno, G. , Regnaut, S. , & Goudet, J. (2005). Detecting the number of clusters of individuals using the software STRUCTURE: A simulation study. Molecular Ecology, 14(8), 2611–2620. 10.1111/j.1365-294X.2005.02553.x 15969739

[ece35937-bib-0023] Excoffier, L. , & Lischer, H. E. L. (2010). Arlequin suite ver 3. 5: A new series of programs to perform population genetics analyses under Linux and Windows. Molecular Ecology Resources, 10, 564–567. 21565059 10.1111/j.1755-0998.2010.02847.x

[ece35937-bib-0024] Falush, D. , Stephens, M. , & Pritchard, J. K. (2003). Inference of population structure using multilocus genotype data: Linked loci and correlated allele frequencies. Genetics, 164(4), 1567–1587. 12930761 10.1093/genetics/164.4.1567 PMC1462648

[ece35937-bib-0025] Falush, D. , Stephens, M. , & Pritchard, J. K. (2007). Inference of population structure using multilocus genotype data: Dominant markers and null alleles. Molecular Ecology Notes, 7(4), 574–578. 10.1111/j.1471-8286.2007.01758.x 18784791 PMC1974779

[ece35937-bib-0026] Ferguson, A. , & Mason, F. M. (1981). Allozyme evidence for reproductively isolated sympatric populations of brown trout *Salmo trutta* . Journal of Fish Biology, 18(6), 629–642.

[ece35937-bib-0027] Ferguson, A. , Reed, T. E. , Cross, T. F. , Mcginnity, P. , & Prodöhl, P. A. (2019). Anadromy, potamodromy and residency in brown trout *Salmo trutta*: The role of genes and the environment. Journal of Fish Biology, 95(3), 1–27. 10.1111/jfb.14005 PMC6771713 31197849

[ece35937-bib-0028] Ferguson, A. , & Taggart, J. B. (1991). Genetic differentiation among the sympatric brown trout (*Salmo trutta*) populations of Lough Melvin, Ireland. Biological Journal of the Linnean Society, 43(January), 221–237. 10.1111/j.1095-8312.1991.tb00595.x

[ece35937-bib-0029] Foldvik, A. , & Kvingedal, E. (2018). Long‐term PIT tag retention rates in Atlantic salmon (*Salmo salar*). Animal Biotelemetry, BioMed Central, 6(3), 4–7. 10.1186/s40317-018-0147-1

[ece35937-bib-0030] Förschler, M. I. , del Val, E. , & Bairlein, F. (2010). Extraordinary high natal philopatry in a migratory passerine. Journal of Ornithology, 151(3), 745–748. 10.1007/s10336-010-0495-y

[ece35937-bib-0031] Fraser, D. J. , Weir, L. K. , Bernatchez, L. , Hansen, M. M. , & Taylor, E. B. (2011). Extent and scale of local adaptation in salmonid fishes: Review and meta‐analysis. Heredity, Nature Publishing Group, 106(3), 404–420. 10.1038/hdy.2010.167 21224881 PMC3131967

[ece35937-bib-0032] Garant, D. , Forde, S. E. , & Hendry, A. P. (2007). The multifarious effects of dispersal and gene flow on contemporary adaptation. Functional Ecology, 21(3), 434–443. 10.1111/j.1365-2435.2006.01228.x

[ece35937-bib-0033] Gomez‐Uchida, D. , Knight, T. W. , & Ruzzante, D. E. (2009). Interaction of landscape and life history attributes on genetic diversity, neutral divergence and gene flow in a pristine community of salmonids. Molecular Ecology, 18(23), 4854–4869. 10.1111/j.1365-294X.2009.04409.x 19878451

[ece35937-bib-0034] Hand, B. K. , Muhlfeld, C. C. , Wade, A. A. , Kovach, R. P. , Whited, D. C. , Narum, S. R. , … Luikart, G. (2016). Climate variables explain neutral and adaptive variation within salmonid metapopulations: The importance of replication in landscape genetics. Molecular Ecology, 25(3), 689–705. 10.1111/mec.13517 26677031

[ece35937-bib-0035] Handeland, S. O. , Imsland, A. K. , & Stefansson, S. O. (2008). The effect of temperature and fish size on growth, feed intake, food conversion efficiency and stomach evacuation rate of Atlantic salmon post‐smolts. Aquaculture, 283, 36–42. 10.1016/j.aquaculture.2008.06.042

[ece35937-bib-0036] Hendry, A. P. , Castric, V. , Kinnison, M. T. , & Quinn, T. P. (2004). The evolution of philopatry and dispersal: Homing versus straying in salmonids In Hendry A. P. & Stearns S. C. (Eds.), Evolution illuminated: Salmon and their relatives (pp. 52–91). Oxford and New York: Oxford University Press.

[ece35937-bib-0037] Hilborn, R. , Quinn, T. P. , Schindler, D. E. , & Rogers, D. E. (2003). Biocomplexity and fisheries sustainability. Proceedings of the National Academy of Sciences, 100(11), 6564–6568. 10.1073/pnas.1037274100 PMC164486 12743372

[ece35937-bib-0038] Hordyk, A. , Ono, K. , Sainsbury, K. , Loneragan, N. R. , & Prince, J. (2014). Some explorations of the life history ratios to describe length composition, spawning‐per‐recruit, and the spawning potential ratio. ICES Journal of Marine Science, 72(1), 204–216. 10.1093/icesjms/fst235

[ece35937-bib-0039] Hubisz, M. J. , Falush, D. , Stephens, M. , & Pritchard, J. K. (2009). Inferring weak population structure with the assistance of sample group information. Molecular Ecology Resources, 9(5), 1322–1332. 10.1111/j.1755-0998.2009.02591.x 21564903 PMC3518025

[ece35937-bib-0040] Jacobs, A. , Hughes, M. R. , Robinson, P. C. , Adams, C. E. , & Elmer, K. R. (2018). The genetic architecture underlying the evolution of a rare piscivorous life history form in brown trout after secondary contact and strong introgression. Genes, 280(9), 1–23. 10.3390/genes9060280 PMC6026935 29857499

[ece35937-bib-0041] Jakobsson, M. , & Rosenberg, N. A. (2007). CLUMPP: A cluster matching and permutation program for dealing with label switching and multimodality in analysis of population structure. Bioinformatics, 23(14), 1801–1806. 10.1093/bioinformatics/btm233 17485429

[ece35937-bib-0042] Jones, O. R. , & Wang, J. (2010). COLONY: A program for parentage and sibship inference from multilocus genotype data. Molecular Ecology Resources, 10(3), 551–555. 10.1111/j.1755-0998.2009.02787.x 21565056

[ece35937-bib-0043] Jonsson, B. , & Jonsson, N. (2001). Polymorphism and speciation in *Arctic charr* . Journal of Fish Biology, 58(3), 605–638. 10.1111/j.1095-8649.2001.tb00518.x

[ece35937-bib-0044] Jonsson, B. , & Jonsson, N. (2014). Naturally and hatchery produced European trout *Salmo trutta*: Do their marine survival and dispersal differ? Journal of Coastal Conservation Planning and Management, 18(2), 79–87. 10.1007/s11852-012-0224-1

[ece35937-bib-0045] Jonsson, B. , & Jonsson, N. (2017). Maternal inheritance influences homing and growth of hybrid offspring between wild and farmed Atlantic salmon. Aquaculture Environment Interactions, 9, 231–238. 10.3354/aei00232

[ece35937-bib-0046] Jonsson, N. , Jonsson, B. , Skurdal, J. , & Hansen, L. P. (1994). Differential response to water current in offspring of inlet and outlet spawning brown trout *Salmo salar* . Journal of Fish Biology, 45, 356–359.

[ece35937-bib-0047] Kamvar, Z. N. , Brooks, J. C. , & Grünwald, N. J. (2015). Novel R tools for analysis of genome‐wide population genetic data with emphasis on clonality. Frontiers in Genetics, 6, 1–10. 10.3389/fgene.2015.00208 26113860 PMC4462096

[ece35937-bib-0048] Kamvar, Z. N. , Tabima, J. F. , & Grünwald, N. J. (2014). Poppr: An R package for genetic analysis of populations with clonal, partially clonal, and/or sexual reproduction. PeerJ, 2, e281. 24688859 10.7717/peerj.281 PMC3961149

[ece35937-bib-0049] Kawecki, T. J. , & Ebert, D. (2004). Conceptual issues in local adaptation. Ecology Letters, 7, 1225–1241. 10.1111/j.1461-0248.2004.00684.x

[ece35937-bib-0050] Kawecki, T. J. , & Holt, R. D. (2002). Evolutionary consequences of asymmetric dispersal rates. The American Naturalist, 160(3), 333–347. 10.1086/341519 18707443

[ece35937-bib-0051] Kaya, C. M. (1991). Rheotactic differentiation between fluvial and lacustrine populations of Arctic grayling (*Thymallus arcticus*) implications for the only remaining indigenous population of fluvial ‘Montana Grayling’. Canadian Journal of Fisheries and Aquatic Science, 48, 53–59. 10.1139/f91-008

[ece35937-bib-0052] Keefer, M. L. , & Caudill, C. C. (2014). Homing and straying by anadromous salmonids: A review of mechanisms and rates. Reviews in Fish Biology and Fisheries, 24(1), 333–368. 10.1007/s11160-013-9334-6

[ece35937-bib-0053] Keenan, K. , Bradley, C. R. , Magee, J. J. , Hynes, R. A. , Kennedy, R. J. , Crozier, W. W. , … Prodöhl, P. A. (2013). Beaufort trout MicroPlex: A high‐throughput multiplex platform comprising 38 informative microsatellite loci for use in resident and anadromous (sea trout) brown trout *Salmo trutta* genetic studies. Journal of Fish Biology, 82(6), 1789–1804. 23731137 10.1111/jfb.12095

[ece35937-bib-0054] Keenan, K. , Mcginnity, P. , Cross, T. F. , Crozier, W. W. , & Prodöhl, P. A. (2013). diveRsity: An R package for the estimation and exploration of population genetics parameters and their associated errors. Methods in Ecology and Evolution, 4(8), 782–788.

[ece35937-bib-0055] Kelso, B. W. , & Northcote, T. G. (1981). Current response of young rainbow trout from inlet and outlet spawning stocks of a British Columbia lake. Verhandlungen Des Internationalen Verein Limnologie, 21, 1214–1221. 10.1080/03680770.1980.11897163

[ece35937-bib-0056] King, R. A. , Hillman, R. , Elsmere, P. , Stockley, B. , & Stevens, J. R. (2016). Investigating patterns of straying and mixed stock exploitation of sea trout, *Salmo trutta*, in rivers sharing an estuary in south‐west England. Fisheries Management and Ecology, 23(5), 376–389. 10.1111/fme.12181

[ece35937-bib-0057] Lesage, L. , Crête, M. , Huot, J. , Dumont, A. , & Ouellet, J. (2000). Seasonal home range size and philopatry in two northern white‐tailed deer populations. Canadian Journal of Zoology, 78, 1930–1940. 10.1139/z00-117

[ece35937-bib-0058] Linløkken, A. N. , Johansen, W. , & Wilson, R. C. (2014). Genetic structure of brown trout, *Salmo trutta*, populations from differently sized tributaries of Lake Mjøsa in south‐east Norway. Fisheries Management and Ecology, 21(6), 515–525. 10.1111/fme.12101

[ece35937-bib-0059] Lobón‐Cerviá, J. , & Sanz, N. (Eds.) (2017). Brown trout: Biology, ecology and management (1st ed.). New York: John Wiley & Sons Ltd.

[ece35937-bib-0060] Mantel, N. (1967). The detection of disease clustering and a generalized regression approach. Cancer Research, 27(1), 2019–2220. 6018555

[ece35937-bib-0061] Markevich, G. , Esin, E. , & Anisimova, L. (2018). Basic description and some notes on the evolution of seven sympatric morphs of Dolly Varden *Salvelinus malma* from the Lake Kronotskoe Basin. Ecology and Evolution, 8(5), 2554–2567. 10.1002/ece3.3806 29531676 PMC5838070

[ece35937-bib-0062] Massa‐Gallucci, A. , Coscia, I. , O'Grady, M. , Kelly‐Quinn, M. , & Mariani, S. (2010). Patterns of genetic structuring in a brown trout (*Salmo trutta* L.) metapopulation. Conservation Genetics, 11(5), 1689–1699. 10.1007/s10592-010-0061-4

[ece35937-bib-0063] McGinnity, P. , de Eyto, E. , Cross, T. F. , Coughlan, J. , Whelan, K. , & Ferguson, A. (2007). Population specific smolt development, migration and maturity schedules in Atlantic salmon in a natural river environment. Aquaculture, 273(2–3), 257–268. 10.1016/j.aquaculture.2007.10.008

[ece35937-bib-0064] McGinnity, P. , Jennings, E. , deEyto, E. , Allott, N. , Samuelsson, P. , Rogan, G. , … Cross, T. (2009). Impact of naturally spawning captive‐bred Atlantic salmon on wild populations: Depressed recruitment and increased risk of climate‐mediated extinction. Proceedings of the Royal Society B: Biological Sciences, 276(1673), 3601–3610. 10.1098/rspb.2009.0799 PMC2817300 19640880

[ece35937-bib-0065] McKeown, N. J. , Hynes, R. A. , Duguid, R. A. , Ferguson, A. , & Prodöhl, P. A. (2010). Phylogeographic structure of brown trout *Salmo trutta* in Britain and Ireland: Glacial refugia, postglacial colonization and origins of sympatric populations. Journal of Fish Biology, 76(2), 319–347. 10.1111/j.1095-8649.2009.02490.x 20738710

[ece35937-bib-0066] Mcphee, M. V. , Whited, D. C. , Kuzishchin, K. V. , & Stanford, J. A. (2014). The effects of riverine physical complexity on anadromy and genetic diversity in steelhead or rainbow trout *Oncorhynchus mykiss* around the Pacific Rim. Journal of Fish Biology, 85(1), 132–150. 10.1111/jfb.12286 24766581

[ece35937-bib-0067] Mobley, K. B. , Granroth‐Wilding, H. , Ellmen, M. , Vähä, J.‐P. , Aykanat, T. , Johnston, S. E. , … Primmer, C. R. (2019). Home ground advantage: Local Atlantic salmon have higher reproductive fitness than dispersers in the wild. Science Advances, 5(2), 1–8. 10.1126/sciadv.aav1112 PMC6392789 30820455

[ece35937-bib-0068] Moreira, A. L. , & Taylor, E. B. (2015). The origin and genetic divergence of ‘black’ kokanee, a novel reproductive ecotype of *Oncorhynchus nerka* . Canadian Journal of Fisheries and Aquatic Sciences, 72(10), 1584–1595. 10.1139/cjfas-2015-0145

[ece35937-bib-0069] Neuheimer, A. B. , & Taggart, C. T. (2007). The growing degree‐day and fish size‐at‐age: The overlooked metric. Canadian Journal of Fisheries and Aquatic Science, 64, 375–385. 10.1139/f07-003

[ece35937-bib-0070] Neville, H. M. , Isaak, D. J. , Dunham, J. B. , Thurow, R. F. , & Rieman, B. E. (2006). Fine‐scale natal homing and localized movement as shaped by sex and spawning habitat in *Chinook salmon*: Insights from spatial autocorrelation analysis of individual genotypes. Molecular Ecology, 15(14), 4589–4602. 10.1111/j.1365-294X.2006.03082.x 17107485

[ece35937-bib-0071] Nevoux, M. , Finstad, B. , Davidsen, J. G. , Finlay, R. , Josset, Q. , Poole, R. , Hojesjo, J. et al (2019). Partial anadromy: A life history strategy of coastal brown trout (*Salmo trutta*, Salmonidae). Fish and Fisheries, 10.1111/faf.12396

[ece35937-bib-0072] Nosil, P. , Egan, S. P. , & Funk, D. J. (2008). Heterogeneous genomic differentiation between walking‐stick ecotypes: ‘Isolation by adaptation’ and multiple roles for divergent selection. Evolution, 62(2), 316–336. 10.1111/j.1558-5646.2007.00299.x 17999721

[ece35937-bib-0073] Olsen, J. B. , Crane, P. A. , Flannery, B. G. , Dunmall, K. , Templin, W. D. , & Wenburg, J. K. (2011). Comparative landscape genetic analysis of three Pacific salmon species from subarctic North America. Conservation Genetics, 12(1), 223–241. 10.1007/s10592-010-0135-3

[ece35937-bib-0074] Orsini, L. , Vanoverbeke, J. , Swillen, I. , Mergeay, J. , & De Meester, L. (2013). Drivers of population genetic differentiation in the wild: Isolation by dispersal limitation, isolation by adaptation and isolation by colonization. Molecular Ecology, 22(24), 5983–5999. 10.1111/mec.12561 24128305

[ece35937-bib-0075] Östergren, J. , & Nilsson, J. (2012). Importance of life‐history and landscape characteristics for genetic structure and genetic diversity of brown trout (*Salmo trutta* L.). Ecology of Freshwater Fish, 21(1), 119–133. 10.1111/j.1600-0633.2011.00529.x

[ece35937-bib-0076] Ozerov, M. Y. , Veselov, A. E. , Lumme, J. , & Primmer, C. R. (2012). ‘Riverscape’ genetics: River characteristics influence the genetic structure and diversity of anadromous and freshwater Atlantic salmon (*Salmo salar*) populations in northwest Russia. Canadian Journal of Fisheries and Aquatic Sciences, 69(12), 1947–1958. 10.1139/f2012-114

[ece35937-bib-0077] Palmé, A. , Laikre, L. , & Ryman, N. (2013). Monitoring reveals two genetically distinct brown trout populations remaining in stable sympatry over 20 years in tiny mountain lakes. Conservation Genetics, 14(4), 795–808. 10.1007/s10592-013-0475-x

[ece35937-bib-0078] Paradis, E. , & Schliep, K. (2018). ape 5.0: An environment for modern phylogenetics and evolutionary analyses in R. Bioinformatics, 35(3), 526–528. 10.1093/bioinformatics/bty633 30016406

[ece35937-bib-0079] Paris, J. R. , King, R. A. , & Stevens, J. R. (2015). Human mining activity across the ages determines the genetic structure of modern brown trout (*Salmo trutta* L.) populations. Evolutionary Applications, 8(6), 573–585. 10.1111/eva.12266 26136823 PMC4479513

[ece35937-bib-0080] Perrier, C. , Guyomard, R. , Bagliniere, J. L. , & Evanno, G. (2011). Determinants of hierarchical genetic structure in Atlantic salmon populations: Environmental factors vs. anthropogenic influences. Molecular Ecology, 20(20), 4231–4245. 10.1111/j.1365-294X.2011.05266.x 21917045

[ece35937-bib-0081] Pringle, J. M. , Blakeslee, A. M. H. , Byers, J. E. , & Roman, J. (2011). Asymmetric dispersal allows an upstream region to control population structure throughout a species' range. Proceedings of the National Academy of Sciences, 108(37), 15288–15293. 10.1073/pnas.1100473108 PMC3174593 21876126

[ece35937-bib-0082] Pritchard, J. K. , Stephens, M. , & Donnelly, P. (2000). Inference of population structure using multilocus genotype data. Genetics, 155(2), 945–959. 10835412 10.1093/genetics/155.2.945 PMC1461096

[ece35937-bib-0083] Pritchard, J. K. , Wen, X. , & Falush, D. (2007). Documentation for structure software: Version 2.2. Software: Practice and Experience, 6(3), 321–326.

[ece35937-bib-0084] Prodöhl, P. A. , Antoniacomi, A. , Bradley, C. , Carlsson, J. , Carvalho, G. R. , Coughlan, J. , … Cross, T. F. (2017). Population genetics and Genetic Stock Identification of anadromous Salmo trutta from the Irish Sea and adjacent areas, using microsatellite DNA loci In Harris G. (Ed.), Sea trout: Management & science (pp. 69–95). Leicestershire, England: Matador Publishing Ltd.

[ece35937-bib-0085] Prodőhl, P. A. , Ferguson, A. , Bradley, C. R. , Ade, R. , Roberts, C. , Keay, E. J. , Costa, A. R. et al (2019). Impacts of acidification on brown trout populations and the contrubution of stocking to population recovery and genetic diversity. Journal of Fish Biology, online Ea, 10.1111/jfb.14054 PMC6852074 31111501

[ece35937-bib-0086] Purdue, J. R. , Smith, M. H. , & Patton, J. C. (2000). Female philopatry and extreme spatial genetic heterogeneity in white‐tailed deer. Journal of Mammalogy, 81(1), 179–185. 10.1644/1545-1542(2000)081<0179:FPAESG>2.0.CO;2

[ece35937-bib-0087] Quinn, T. P. , Stewart, I. J. , & Boatright, C. P. (2006). Experimental evidence of homing to site of incubation by mature sockeye salmon, *Oncorhynchus nerka* . Animal Behaviour, 72(4), 941–949. 10.1016/j.anbehav.2006.03.003

[ece35937-bib-0088] Quinn, T. P. , & Tallman, R. F. (1987). Seasonal environmental predictability and homing in riverine fishes. Environmental Biology of Fishes, 18(2), 155–159. 10.1007/BF00002604

[ece35937-bib-0089] R Core Team . (2018). R: A language and environment for statistical computing. Vienna, Austria: R Foundation for Statistical Computing.

[ece35937-bib-0090] Raleigh, R. F. (1967). Genetic control in the lakeward migrations of sockeye salmon (*Oncorhynchus nerka*) fry. Journal of the Fisheries Research Board of Canada, 24(12), 2613–2622. 10.1139/f67-209

[ece35937-bib-0091] Raleigh, R. F. (1971). Innate control of migrations of salmon and trout fry from natal gravels to rearing areas. Ecology, 52(2), 291–297. 10.2307/1934587

[ece35937-bib-0092] Raleigh, R. F. , & Chapman, D. W. (1971). Genetic control in lakeward migrations of cutthroat trout fry. Transactions of the American Fisheries Society, 1, 33–40. 10.1577/1548-8659(1971)100<33:GCILMO>2.0.CO;2

[ece35937-bib-0093] Reynolds, J. , Weir, B. S. , & Cockerham, C. C. (1983). Estimation of the coancestry coefficient: Basis for a short‐term genetic distance. Genetics, 105(3), 767–779. 17246175 10.1093/genetics/105.3.767 PMC1202185

[ece35937-bib-0094] Richardson, J. L. , Urban, M. C. , Bolnick, D. I. , & Skelly, D. K. (2014). Microgeographic adaptation and the spatial scale of evolution. Trends in Ecology and Evolution, 29(3), 165–176. 10.1016/j.tree.2014.01.002 24560373

[ece35937-bib-0095] Rodríguez‐Ramilo, S. T. , Toro, M. A. , Wang, J. , & Fernández, J. (2014). Improving the inference of population genetic structure in the presence of related individuals. Genetics Research, 96(July), e003. 25022872 10.1017/S0016672314000068 PMC7044976

[ece35937-bib-0096] Rodríguez‐Ramilo, S. T. , & Wang, J. (2012). The effect of close relatives on unsupervised Bayesian clustering algorithms in population genetic structure analysis. Molecular Ecology Resources, 12(5), 873–884. 10.1111/j.1755-0998.2012.03156.x 22639868

[ece35937-bib-0097] Rousset, F. (2008). A complete reimplementation of the Genepop software for Windows and Linux. Molecular Ecology Resources, 8, 103–106. 21585727 10.1111/j.1471-8286.2007.01931.x

[ece35937-bib-0098] Sanmartı, I. , Wanntorp, L. , & Winkworth, R. C. (2007). West Wind Drift revisited: Testing for directional dispersal in the Southern Hemisphere using event‐based tree fitting. Journal of Biogeography, 34, 398–416. 10.1111/j.1365-2699.2006.01655.x

[ece35937-bib-0099] Schindler, D. E. , Armstrong, J. B. , & Reed, T. E. (2015). The portfolio concept in ecology and evolution. Frontiers in Ecology and the Environment, 13(5), 257–263. 10.1890/140275

[ece35937-bib-0100] Slatkin, M. (1995). A measure of population subdivision based on microsatellite allele frequencies. Genetics, 139, 457–462. 7705646 10.1093/genetics/139.1.457 PMC1206343

[ece35937-bib-0101] Stewart, I. J. , Quinn, T. P. , & Bentzen, P. (2003). Evidence for fine‐scale natal homing among island beach spawning sockeye salmon, *Oncorhynchus nerka* . Environmental Biology of Fishes, 67(1), 77–85.

[ece35937-bib-0102] Storch, L. S. , & Pringle, J. M. (2018). A downstream drift into chaos: Asymmetric dispersal in a classic density dependent population model. Theoretical Population Biology, Elsevier Inc., 9, 1–9. 10.1016/j.tpb.2018.04.003 29729945

[ece35937-bib-0103] Sundqvist, L. , Keenan, K. , Zackrisson, M. , Prodöhl, P. , & Kleinhans, D. (2016). Directional genetic differentiation and relative migration. Ecology and Evolution, 6(11), 3461–3475. 10.1002/ece3.2096 27127613 PMC4842207

[ece35937-bib-0104] Taggart, J. B. , McLaren, I. S. , Hay, D. W. , Webb, J. H. , & Youngson, A. F. (2001). Spawning success in Atlantic salmon (*Salmo salar* L.): A long‐term DNA profiling‐based study conducted in a natural stream. Molecular Ecology, 10, 1047–1060. 10.1046/j.1365-294X.2001.01254.x 11348510

[ece35937-bib-0105] Taylor, P. , Meyer, K. A. , High, B. , Gastelecutto, N. , Mamer, E. R. J. , Steven, F. , Meyer, K. A. et al (2011). Retention of passive integrated transponder tags in stream‐dwelling rainbow trout. North American Journal of Fisheries Management, 31, 236–239. 10.1080/02755947.2011.572799

[ece35937-bib-0106] Taylors, E. B. , & Bentzent, P. (1993). Molecular genetic evidence for reproductive isolation between sympatric populations of smelt Osmerus in Lake Utopia, south‐western New Brunswick, Canada. Molecular Ecology, 2, 345–357. 10.1111/j.1365-294X.1993.tb00028.x

[ece35937-bib-0107] Tonteri, A. , Veselov, A. J. , Titov, S. , Lumme, J. , & Primmer, C. R. (2007). The effect of migratory behaviour on genetic diversity and population divergence: A comparison of anadromous and freshwater Atlantic salmon *Salmo salar* . Journal of Fish Biology, 70(supplement C), 381–398. 10.1111/j.1095-8649.2007.01519.x

[ece35937-bib-0108] Torterotot, J. , Perrier, C. , Bergeron, N. E. , & Bernatchez, L. (2014). Influence of forest road culverts and waterfalls on the fine‐scale distribution of brook trout genetic diversity in a boreal watershed. Transactions of the American Fisheries Society, 143, 1577–1591. 10.1080/00028487.2014.952449

[ece35937-bib-0109] Vähä, J. P. , Erkinaro, J. , Niemelä, E. , & Primmer, C. R. (2007). Life‐history and habitat features influence the within‐river genetic structure of Atlantic salmon. Molecular Ecology, 16(13), 2638–2654. 10.1111/j.1365-294X.2007.03329.x 17594436

[ece35937-bib-0110] Van Oosterhout, C. , Hutchinson, W. , Wills, D. , & Shipley, P. (2004). MICRO-CHECKER: software for identifying and correcting genotyping errors in microsatellite data. Molecular Ecology Notes, 4(3), 535–538.

[ece35937-bib-0111] Waples, R. S. , & Do, C. (2010). Linkage disequilibrium estimates of contemporary Ne using highly variable genetic markers: A largely untapped resource for applied conservation and evolution. Evolutionary Applications, 3(3), 244–262. 10.1111/j.1752-4571.2009.00104.x 25567922 PMC3352464

[ece35937-bib-0112] Waples, R. S. , & Gaggiotti, O. (2006). What is a population? An empirical evaluation of some genetic methods for identifying the number of gene pools and their degree of connectivity. Molecular Ecology, 15, 1419–1439. 10.1111/j.1365-294X.2006.02890.x 16629801

[ece35937-bib-0113] Ward, R. D. , Woodwark, M. , & Skibinski, D. O. F. (1994). A comparison of genetic diversity levels in marine, freshwater, and anadromous fishes. Journal of Fish Biology, 44(2), 213–232. 10.1111/j.1095-8649.1994.tb01200.x

[ece35937-bib-0114] Watkinson, A. R. , & Sutherland, W. J. (1995). Sources, sinks and pseudo‐sinks. Journal of Animal Ecology, 64(1), 126–130. 10.2307/5833

[ece35937-bib-0115] Weatherhead, P. J. , & Forbes, M. R. L. (1994). Natal philopatry in passerine birds: Genetic or ecological influences? Behavioral Ecology, 5(4), 426–433. 10.1093/beheco/5.4.426

[ece35937-bib-0116] Whelan, K. F. , Poole, W. R. , McGinnity, P. , Rogan, G. , & Cotter, D. (1998). The Burrishoole System. Studies of Irish Rivers and Lakes, Essays on the Occasion of the XXVII Congress of Societas Internationalis Limnologiae (SIL), Dublin, pp. 191–212.

[ece35937-bib-0117] Wilson, G. A. , & Rannala, B. (2003). Bayesian inference of recent migration rates using multilocus genotypes. Genetics, 163(3), 1177–1191. 12663554 10.1093/genetics/163.3.1177 PMC1462502

[ece35937-bib-0118] Winkler, D. W. , Wrege, P. H. , Allen, P. E. , Kast, T. L. , Senesac, P. , Wasson, M. F. , … Sullivan, P. J. (2006). Breeding dispersal and philopatry in the tree swallow. The Condor, 106(4), 768–776. 10.1093/condor/106.4.768

